# Exploring white matter microstructural alterations in mild cognitive impairment: a multimodal diffusion MRI investigation utilizing diffusion kurtosis and free-water imaging

**DOI:** 10.3389/fnins.2024.1440653

**Published:** 2024-08-07

**Authors:** Megan R. Nelson, Elizabeth G. Keeling, Ashley M. Stokes, Maurizio Bergamino

**Affiliations:** ^1^Division of Neuroimaging Research, Barrow Neurological Institute, Phoenix, AZ, United States; ^2^School of Life Sciences, Arizona State University, Tempe, AZ, United States

**Keywords:** mild cognitive impairment, dementia, free-water DTI, diffusion kurtosis imaging, mean signal diffusion kurtosis imaging

## Abstract

**Background:**

Mild Cognitive Impairment (MCI) is a transitional stage from normal aging to dementia, characterized by noticeable changes in cognitive function that do not significantly impact daily life. Diffusion MRI (dMRI) plays a crucial role in understanding MCI by assessing white matter integrity and revealing early signs of axonal degeneration and myelin breakdown before cognitive symptoms appear.

**Methods:**

This study utilized the Alzheimer’s Disease Neuroimaging Initiative (ADNI) database to compare white matter microstructure in individuals with MCI to cognitively normal (CN) individuals, employing advanced dMRI techniques such as diffusion kurtosis imaging (DKI), mean signal diffusion kurtosis imaging (MSDKI), and free water imaging (FWI).

**Results:**

Analyzing data from 55 CN subjects and 46 individuals with MCI, this study found significant differences in white matter integrity, particularly in free water levels and kurtosis values, suggesting neuroinflammatory responses and microstructural integrity disruption in MCI. Moreover, negative correlations between Mini-Mental State Examination (MMSE) scores and free water levels in the brain within the MCI group point to the potential of these measures as early biomarkers for cognitive impairment.

**Conclusion:**

In conclusion, this study demonstrates how a multimodal advanced diffusion imaging approach can uncover early microstructural changes in MCI, offering insights into the neurobiological mechanisms behind cognitive decline.

## 1 Introduction

Mild cognitive impairment (MCI) is characterized by changes in cognitive abilities that exceed what might be expected from normal cognitive aging but do not significantly interfere with daily activities (Anand and Schoo, 2024). Factors such as genetics, diabetes, depression, stroke, and other medical conditions can contribute to the development of MCI (Roberts and Knopman, 2013). Neuropathological changes due to Alzheimer’s disease (AD) pathology, including amyloid-beta plaques, tau tangles, and neurodegeneration, may also contribute to the development of MCI. While MCI is a risk factor for dementia, not all individuals with MCI progress to dementia; some remain stable, and others may even revert to normal cognitive function.

Several neuroimaging techniques have emerged as valuable tools for investigating the neuropathological changes associated with MCI in recent years (Yanhong et al., 2013; Yin et al., 2013). Among these techniques, diffusion MRI (dMRI) is used to study the microstructural alterations in brain tissue. By measuring the diffusion of water molecules in the brain, dMRI provides insights into the integrity of white matter tracts and the organization of neuronal connections (Le Bihan, 2014; Mueller et al., 2015), which is often compromised in individuals with MCI. Disruptions in white matter microstructure, such as axonal degeneration and myelin breakdown, can precede the onset of cognitive symptoms and serve as early biomarkers of neurodegenerative processes (Power et al., 2019). Additionally, dMRI allows for the investigation of structural connectivity networks in the brain (Zhang et al., 2022), shedding light on the degeneration of functional pathways associated with cognitive decline in MCI.

Diffusion tensor imaging (DTI) is a technique widely employed in dMRI studies, owing to its proficiency in assessing white matter microstructure and inferring fiber tractography. DTI metrics like fractional anisotropy (FA) and tractography (Tae et al., 2018) enable the identification of network topology changes and specific brain regions particularly vulnerable to degeneration. However, DTI-derived metrics may provide ambiguous or misleading data in areas with crossing fibers or complex tissue microenvironments, thus hindering the accurate assessment of white matter integrity (Coutu et al., 2014). Advanced dMRI methods, such as diffusion kurtosis imaging (DKI) (Jensen et al., 2005; Jensen and Helpern, 2010; Falangola et al., 2013) and free-water imaging (FWI) (Pasternak et al., 2009), have emerged as robust alternatives to conventional DTI. These approaches address the limitations of DTI by modeling non-Gaussian diffusion processes associated with intricate fiber geometries and accommodating various tissue compartments (Jensen et al., 2005; Jensen and Helpern, 2010; Steven et al., 2014), such as cerebrospinal fluid (CSF) and extracellular free water (Pierpaoli et al., 1996).

DKI captures the complex diffusion patterns exhibited by biological tissues more accurately than DTI by incorporating higher-order diffusion moments, such as kurtosis, which provides insights into tissue microstructural complexity (Jensen et al., 2005; Jensen and Helpern, 2010). Lower diffusion kurtosis values indicate less constrained diffusion, which may correlate with neuronal loss or other pathological alterations (Arab et al., 2018). FWI accounts for the influence of free water contamination by estimating the volume fraction of extracellular free water (*f*) within brain tissue. FWI enables the derivation of DTI metrics corrected for this confounding factor, thus enhancing the specificity of white matter assessments (Pasternak et al., 2009; Bergamino et al., 2017, 2021). DKI and FWI have been used in studies investigating MCI and dementia, yielding significant findings that contribute to the understanding of these conditions (Dumont et al., 2019; Bergamino et al., 2021, 2024; Chu et al., 2022).

In addition to diffusion changes, white matter hyperintensities (WMH) are commonly observed on MRI scans of elderly individuals and are biomarkers of cerebral small vessel disease (Kynast et al., 2018). WMH can disrupt white matter tracts, leading to impaired communication between brain regions (Garnier-Crussard et al., 2023). Previous studies have found that higher WMH volume increases the risk of developing MCI and dementia, indicating that WMH may serve as a biomarker for cognitive decline (Debette and Markus, 2010). For these reasons, WMHs were included as a covariate to help control for its confounding effects, isolating the impact between white matter tracts and cognitive decline in MCI.

This study assessed white matter microstructural differences between a cohort of individuals with MCI and an age-matched group of cognitively normal individuals (CN), utilizing data from the Alzheimer’s Disease Neuroimaging Initiative (ADNI) database (Petersen et al., 2010).^1^ This study utilized three distinct dMRI techniques—free-water DTI, kurtosis imaging, and mean signal diffusion kurtosis imaging (MSDKI)—to investigate MCI. To our knowledge, no prior study has integrated both free-water DTI and kurtosis imaging in MCI research, nor has the MSDKI model been applied in this context. This multi-modal approach enables a comprehensive analysis of white matter microstructural alterations, offering insights that single-technique studies may overlook. Addressing the extracellular free-water component and capturing non-Gaussian diffusion behavior will achieve a more precise characterization of the tissue microstructure heterogeneity associated with MCI. Moreover, voxel-based correlations between the Mini-Mental State Exam (MMSE) scores (Arevalo-Rodriguez et al., 2015) and diffusion metrics were assessed within the MCI group.

## 2 Methods

### 2.1 Participants

All data were downloaded from the ADNI database (Petersen et al., 2010). Inclusion criteria included the designation of CN or MCI and the availability of anatomical and multi-shell dMRI data. Subjects missing the magnetization-prepared rapid gradient-echo (MPRAGE) or T2-weighted fluid-attenuated inversion recovery (T2-FLAIR) images were excluded. Fifty-five CN subjects (39 females; age (standard deviation, SD) = 76.1 (7.0) years) and 46 MCI individuals (16 females age = 74.2 (7.6) years) were included in this study.

Mini-Mental State Examination (MMSE) scores were available for all 55 CN individuals and 45 subjects with MCI. The primary function of the MMSE is to assess cognitive abilities such as orientation, memory, attention, language skills, and visuospatial abilities. Scoring on the MMSE ranges from 0 to 30 points, with a score of 25 or higher considered normal. Scores below 24 may indicate possible cognitive impairment (Arevalo-Rodriguez et al., 2015). Additional cognitive assessments administered to both groups and available in the ADNI dataset included the Geriatric Depression Scale (GDS), a screening tool to evaluate depressive symptoms in older adults (Yesavage et al., 1982); the Functional Activities Questionnaire (FAQ), designed to assess instrumental activities of daily living, particularly in older adults with cognitive impairment (Marshall et al., 2015); and the Clinical Dementia Rating (CDR), a tool used to stage dementia severity and track changes in cognitive function longitudinally (Morris, 1993).

Apolipoprotein E (ApoE) status is a genetic risk factor implicated in AD risk. Patients with the e4 allele show a higher risk of developing MCI and AD, with the additive risk associated with homozygous e4/e4 alleles (Liu et al., 2013). On the other hand, the e2 allele is protective for AD risk. ApoE status and complete demographic and clinical characteristics of the study participants are shown in Table 1.

**Table 1:** Complete subject characteristics.

Group	N (#F)	Age (SD) year	MMSE [available]	GDS [available]	FAQ (total) [available]	Global CDR [available]
CN	55 (39)	76.1 (7.0)	29.04 (1.43) [55]	0.93 (1.75) [55]	0.39 (1.57) [54]	0.07 (0.20) [53]
MCI	46 (16)^*^	74.2 (7.6)	27.36 (3.96) [45]	2.00 (2.12) [46]	3.65 (6.71) [46]	0.40 (0.36) [46]
*Shapiro–Wilk*		*W* = 0.987; *p* = 0.452	*W* = 0.528; *p* < 0.001	*W* = 0.727; *p* < 0.001	*W* = 0.438; *p* < 0.001	*W* = 0.641; *p* < 0.001
*t-test*		*t* = −1.314; *p* = 0.192	–	–	–	–
*Mann–Whitney U test*		–	*W* = 1,668; *p* = 0.002	*W* = 791.5; p < 0.001	*W* = 649.5; *p* < 0.001	*W* = 515.5; *p* < 0.001
Motion/outliers		ABS motion (mm)	REL. motion (mm)	Outliers (%)		
CN		0.76 (0.32)	0.56 (0.18)	0.37 (0.18)		
MCI		0.94 (0.66)	0.66 (0.32)	0.42 (0.27)		
*Shapiro–Wilk*		*W* = 0.672; *p* < 0.001	*W* = 0.799; *p* < 0.001	*W* = 0.844; *p* < 0.001		
*Mann–Whitney U test*		W = 1,029; *p* = 0.108	W = 1,080; *p* = 0.208	W = 1,167; *p* = 0.506		
Included in the final analysis						
CN	55 (39)	76.1 (7.0)	29.04 (1.43) [55]	0.93 (1.75) [55]	0.39 (1.57) [54]	0.07 (0.20) [53]
MCI	45 (15)	74.4 (7.6)	27.34 (4.00) [44]	2.02 (2.14) [45]	3.73 (6.77) [45]	0.41 (0.36) [45]
*Shapiro–Wilk*		*W* = 0.986; *p* = 0.450	*W* = 0.528; p < 0.001	*W* = 728; p < 0.001	*W* = 0.440; p < 0.001	*W* = 0.643; p < 0.001
*t-*test		*t* = −1.162; *p* = 0.248	–	–	–	–
*Mann–Whitney U test*		–	*W* = 1,621; *p* = 0.003	*W* = 755; *p* < 0.001	*W* = 620; *p* < 0.001	*W* = 486; *p* < 0.001
Motion/Outliers		ABS motion (mm)	REL. motion (mm)	Outliers (%)		
CN		0.76 (0.32)	0.56 (0.18)	0.37 (0.18)		
MCI		0.86 (0.40)	0.66 (0.32)	0.42 (0.28)		
*Shapiro–Wilk*		*W* = 0.881; *p* < 0.001	*W* = 0.800; *p* < 0.001	*W* = 0.844; *p* < 0.001		
*Mann–Whitney U test*		*W* = 1,028; *p* = 0.150	*W* = 1,054; *p* = 0.205	*W* = 1,145; *p* = 0.524		
APO-E	CN (#54)	MCI (#44)				
E2E3	6	5				
E3E3	32	18				
E3E4	12	18				
E4E4	4	3				

^*^One female removed due to motion (with no APO-E data). SD, standard deviation; MMSE, Mini-Mental State Examination; GDS, Geriatric Depression Scale; CDR, Clinical Dementia Rating; FAQ, Functional Activities Questionnaire; APO-E, Apolipoprotein E.

### 2.2 MRI acquisition

The dMRI data were downloaded from the ADNI database and acquired on a 3 Tesla Siemens Medical Systems Prisma scanner. All data were obtained from the ADNI-3 cohort, which is currently the only dataset within ADNI that includes multi-shell dMRI acquisitions. Each participant was scanned using an MPRAGE sequence (1.2 mm slices thick; 1.0 × 1.0 mm^2^ in-plane resolution, 256 × 256 matrix, TR: 8.82 s, TE: 3.162 ms, TI: 3.162 ms, flip angle: 9°) and a T2-FLAIR sequence (5 mm slices thick; 0.86 × 0.86 mm^2^ in-plane resolution, 256 × 237 matrix, TR: 9.0 s, TE: 90.0 ms, flip angle: 90°).

The dMRI protocol employed a multi-shell acquisition and utilized spin-echo diffusion-weighted echo-planar imaging with a TR of 3,400 ms, an echo time (TE) of 71 ms, multi-band = 3, and a voxel size of 2.0 × 2.0 × 2.0 mm^3^. The acquisition included a matrix size of 116 × 116, 81 slices, 127 gradient directions with b-values of 500, 1,000 and 2000 s/mm^2^, and 11 b = 0 (b0) images. Additional acquisition information can be found on the ADNI webpage (see footnote 1).

### 2.3 Data pre-processing - dMRI

DICOM data were downloaded from the ADNI database and converted into NIFTI format using the *dcm2niix* tool (available at: https://github.com/rordenlab/dcm2niix). NIFTI data were processed through a series of applications: Mrtrix3 (Tournier et al., 2019),^3^ FMRIB Software Library (FSL) version 6.0 (Jenkinson et al., 2012), and Advanced Normalization Tools (ANTs).^4^ The preprocessing steps for dMRI data included noise reduction using *dwidenoise* (Veraart et al., 2016) (MRtrix3), along with procedures for aligning the images and correcting for eddy currents using *eddy* (Andersson and Sotiropoulos, 2016) (FSL). The eddy QC tools were employed to evaluate the quality of the dMRI data. Instances of signal loss resulting from subject movement coinciding with diffusion encoding were identified, and the affected slices were replaced with predictions generated through a Gaussian process. The quality control criteria for this study were established as an average absolute volume-to-volume head motion value of less than 3 mm or fewer than 5% of total outliers. The brain extraction from the b0 images was performed by *dwi2mask* (Mrtrix3). Following this step, all preprocessed dMRI images were uniformly rescaled to a voxel size of 1.25 mm using *mrgrid* (Mrtrix3). All b0 images were used to create a group-wise template by ANTs. This template was used as a ‘standard’ space for all statistical analyses. For the visualization of significant clusters and the calculation of their volumes within each white matter area, the b0 group-wise template was normalized to the MNI b0 standard image (1x1x1 mm voxel size) using the ANTs Symmetric Image Normalization (SyN) algorithm.

### 2.4 Free water algorithm

The free water elimination model aims to mitigate the negative impact of CSF partial volume effects on diffusion measurements (Hoy et al., 2014). This model can offer a more nuanced understanding of brain tissue by discerning between freely moving water molecules and those that are hindered or restricted. In this study, the computation of FWI-related metrics, including fractional anisotropy (fw-FA) and the fw index (*f*), was conducted using the DIPY^5^ algorithm (*fwdti.FreeWaterTensorModel* class object and *fwdtimodel.fit* function) for multi-shell dMRI through an in-house Python script.

### 2.5 Diffusion kurtosis imaging (DKI)

DKI provides additional information about the non-Gaussian diffusion of water molecules in biological tissues (Jensen et al., 2005; Jensen and Helpern, 2010). Using kurtosis metrics, DKI quantifies the degree of deviation from a Gaussian distribution, an assumption of standard DTI. This model can offer insights into the microstructural complexity of tissues beyond what DTI can provide through the kurtosis coefficient (K), which reflects the deviation from Gaussian diffusion. This study utilized two DKI metrics: the mean kurtosis tensor (MKT) and the kurtosis fractional anisotropy (KFA), which depend solely on the kurtosis tensor. Additionally, standard DKI metrics, including mean kurtosis (MK), radial kurtosis (RK), and axial kurtosis (AK), were analyzed. All DKI metrics were computed using DIPY’s *dki.DiffusionKurtosisModel* class object and the *dkimodel.fit* function via an in-house Python script.

### 2.6 Mean signal diffusion kurtosis imaging (MSDKI)

An essential limitation of DKI is that its measurements are susceptible to noise and image artifacts. For instance, due to low radial diffusivity, standard kurtosis estimates in regions with well-aligned voxels may be corrupted by implausibly low or negative values. To overcome this issue, the MSDKI model can be used (Henriques et al., 2021). This model simplifies the DKI approach by averaging the signal intensities over all diffusion directions for each b-value before calculating the kurtosis. The averaging process reduces the influence of directional variability in water diffusion, yielding a scalar metric that reflects the average diffusion kurtosis across all directions. The mean signal approach aims to simplify the complexity and computational demand of DKI.

MSDKI has several advantages, including reducing data processing and analysis complexity. Averaging signals across directions can mitigate the impact of noise, potentially improving measurement reliability, especially in high b-value ranges. However, by averaging signals, MSDKI sacrifices information on directional diffusion anisotropy, which is essential for understanding the orientation of the tissues inside the white matter.

In this study, the mean signal diffusion (MSD) and the mean signal kurtosis (MSK) metrics were computed using DIPY’s *MeanDiffusionKurtosisModel* class and the *msdki_model.fit* function via an in-house Python script. The MSD metric represents the average diffusion coefficient across all directions within a voxel. It provides insights into the microstructural environment of tissues, particularly in areas with complex architecture. A lower MSD value may indicate denser or more restricted tissue environments, while higher values suggest more free water movement, potentially reflecting damaged tissue. The MSK is a metric that quantitatively describes the degree of non-Gaussian diffusion of water molecules in brain tissue, averaged over all diffusion directions. MSK provides a scalar value reflecting the average excess kurtosis of water diffusion, indicating the complexity and heterogeneity of tissue microstructure without emphasizing diffusion directionality.

### 2.7 White matter hyperintensity (WMH)

WMH are areas of abnormal signal intensity within the white matter, typically visualized on T2-weighted or FLAIR MRI sequences. Automated segmentation of WMH volume was performed with the Lesion Segmentation Tool (LST version 3.0.0 as implemented in SPM12) within MATLAB v. 2023b using T2-weighted FLAIR and MPRAGE images.

In this study, FMRIB’s Automated Segmentation Tool (FAST) (Zhang et al., 2001) segmented the brain into three distinct tissue types: gray matter, white matter, and cerebrospinal fluid (CSF). The total volumes of gray matter, white matter, and CSF were summed to determine the intracranial volume. The volume of white matter hyperintensities (WMH) was then normalized by the intracranial volume. The normalized volumes of the WMH areas were included as covariates for all statistical analyses.

### 2.8 Statistical analyses

The demographic and clinical characteristics, including age, MMSE, GDS, Global CDR, FAQ, motion, and outliers, are presented as means and standard deviations for each group. Differences in age between groups were examined using a Student’s t-test, confirmed by the Shapiro–Wilk (SW) test for normality (*W* = 0.987, *p_SW_* = 0.452). For cognitive assessments (MMSE, GDS, CDR, and FAQ), differences were analyzed using the Mann–Whitney U test due to non-normality (*p_SW_* ≤ 0.001 for all assessments). Group differences in motion and outliers were also analyzed using the Mann–Whitney U test due to non-normality (*p_SW_* < 0.001 for all motion/outlier measurements).

All diffusion metrics computed for this study were coregistered to the group-wise template space by an ANTs SyN coregistration algorithm. The voxel-based analysis (between groups and correlations with MMSE) was restricted to voxels within the combined masks derived from the ICBM-DTI-81 white-matter labels atlas and the JHU white-matter tractography atlas. Clusters showing statistically significant differences across groups were annotated according to the JHU DTI-based white-matter atlases (Wakana et al., 2007; Hua et al., 2008).

A Student’s t-test with a linear model function was employed to assess voxel-based differences across groups in all diffusion-related metrics. To mitigate potential confounding effects, the covariates of age, sex, WMH normalized volume, and average absolute motion were incorporated into the model. Voxel-based correlations between all diffusion metrics and MMSE scores were computed using a linear model with age, sex, WMH normalized volume, and average absolute motion as covariates. Analyses were performed with an in-house R (version 3.6.3) script and RStudio (version 1.3.1093).

The Threshold-Free Cluster Enhancement (TFCE) method was utilized to ensure robust clustering while avoiding arbitrary thresholding and addressing multiple comparisons (Smith and Nichols, 2009). Additionally, a Family-Wise Error (FWE) correction at the 0.05 level using the Benjamini-Hochberg procedure (FDR < 0.05) was applied. Effect sizes were calculated for all analyses using Hedges’ g, where |g| ≥ 0.61 indicated a large effect size for voxel-based differences between groups (*α* = 0.05, power = 0.85), and Spearman’s correlation coefficients with |ρ| > 0.44 as large effects (*α* = 0.05, power = 0.85) for correlations with MMSE.

## 3 Results

One female MCI participant was removed from the final analysis due to excessive motion (absolute motion = 4.50 mm).

Among the final participants, no statistical differences between groups were found for age (*t* = −1.162; *p* = 0.248). However, statistical differences were found in all cognitive tests (MMSE: *W* = 1,621; *p* = 0.003; GDS: *W* = 755; *p* < 0.001; FAQ: *W* = 620; *p* < 0.001; Global CDR: *W* = 486; *p* < 0.001).

No statistical differences were found across the groups in absolute motion (W = 1,028; *p* = 0.150), relative motion (*W* = 1,054; *p =* 0.205), and total outliers (*W* = 1,145; *p =* 0.524). Additionally, there were no significant differences in WMH normalized volume between CN and MCI groups (*t* = 0.454; *p* = 0.651). Statistical results for all cognitive scores, motion, outliers, and APOE status are reported in Table 1.

### 3.1 FWI results

**Figure 1:**
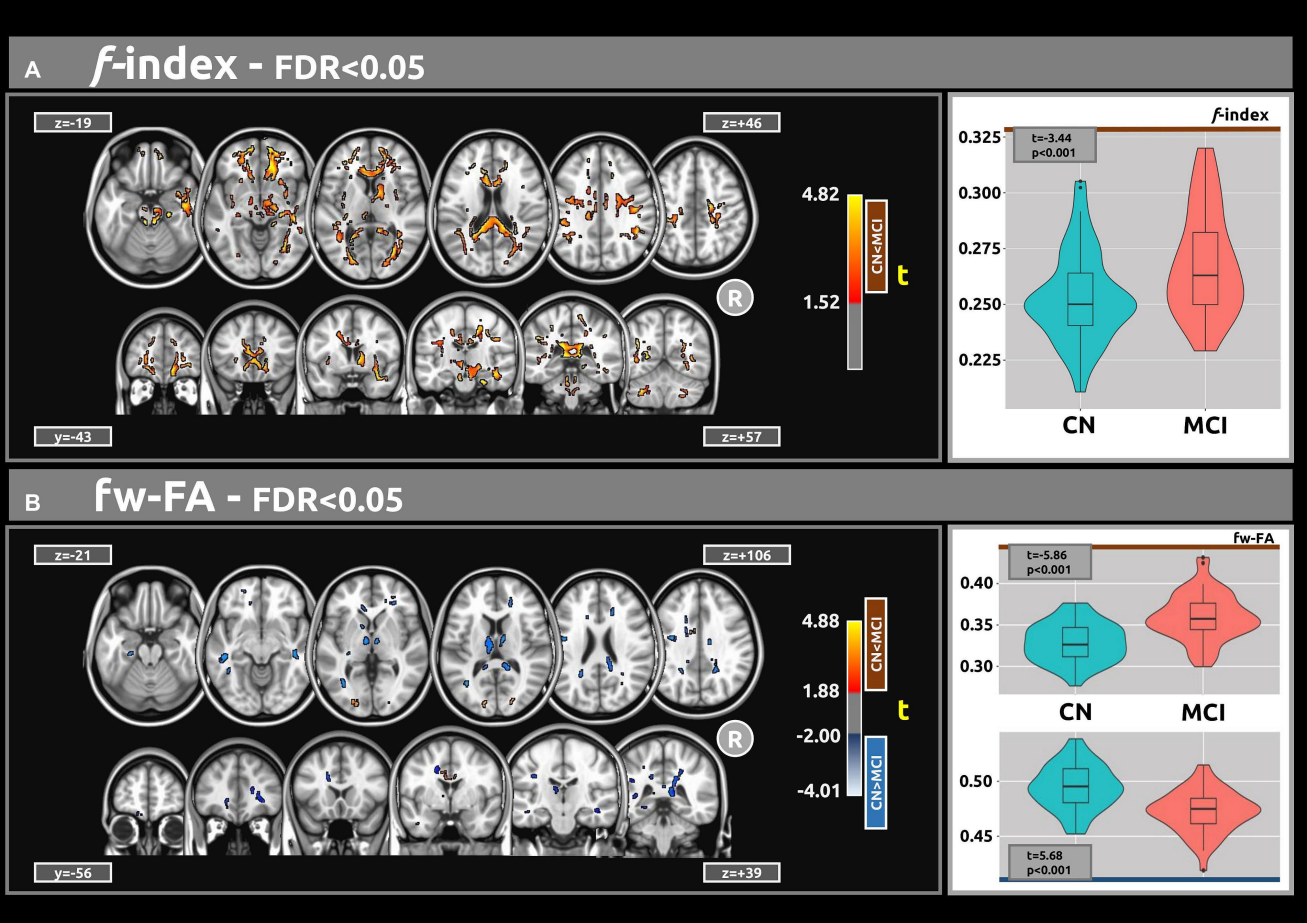
The voxel-based analyses of FWI metrics (*t*-value at FDR < 0.05) are shown in both axial and coronal views. **(A)** Compared with CN individuals, higher values of the f index were observed in the MCI group. **(B)** Group differences were also found for the fw-FA metric. Plots illustrate the mean values within the significant clusters.

Figure 1A illustrates the statistical voxel-based differences between groups for the *f* index. The MCI group exhibited elevated free water levels compared to the CN group. Extensive clusters with |g| > 0.61 were identified in various white matter regions, including the corpus callosum, right cerebral peduncle, right sagittal stratum, and right uncinate fasciculus. Table 2A provides details on the volume, mean t-values, and maximum effect sizes for the clusters with |g| > 0.61. Complete results for the *f* index, including large, medium, and small effect sizes, can be found in Supplementary Table S1.

**Table 2:** Results for fw-DTI (**(A)** f index and **(B)** fw-FA).

fw-DTI			
(A)	*f* index		
	CN < MCI		
	*t*-stats		Effect-size
	Vol (%)	<t>	g_**max**_
JHU Atlas			
Anterior thalamic radiation L	9.02	2.202	0.827
Anterior thalamic radiation R	19.04	2.146	0.810
Cortical spinal tract L	6.25	2.002	0.631
Cortical spinal tract R	17.03	1.990	0.662
Cingulum cingulate gyrus L	12.71	2.018	0.762
Cingulum cingulate gyrus R	7.43	2.208	0.680
Cingulum hippocampus R	21.62	2.563	0.798
Forceps major	21.15	2.266	0.735
Forceps minor	21.69	2.403	0.877
Inferior fronto-occipital fasciculus L	9.29	2.109	0.761
Inferior fronto-occipital fasciculus R	23.84	2.220	0.858
Inferior longitudinal fasciculus L	5.06	2.006	0.845
Inferior longitudinal fasciculus R	28.33	2.305	0.795
Superior longitudinal fasciculus L	9.00	2.097	0.905
Superior longitudinal fasciculus R	7.93	2.013	0.674
Uncinate fasciculus L	9.77	2.146	0.793
Uncinate fasciculus R	33.49	2.353	0.861
Superior longitudinal fasciculus temporal L	11.96	1.948	0.785
Superior longitudinal fasciculus temporal R	9.51	1.926	0.674
ICBM81 Atlas			
Genu of corpus callosum	43.78	2.378	0.825
Splenium of corpus callosum	46.75	2.394	0.735
Superior cerebellar peduncle R	25.00	2.465	0.646
Cerebral peduncle R	56.23	2.042	0.662
Anterior limb of internal capsule R	17.94	2.224	0.629
Anterior corona radiata R	18.32	2.332	0.858
Anterior corona radiata L	4.82	2.096	0.672
Posterior thalamic radiation R	24.85	2.204	0.622
Sagittal stratum R	40.31	2.383	0.757
Cingulum (cingulate gyrus) L	18.36	1.979	0.762
Cingulum (hippocampus) R	43.93	2.634	0.798
Superior longitudinal fasciculus L	17.11	1.915	0.690
Uncinate fasciculus R	74.21	2.388	0.614

(B)	fw-FA					
	CN < MCI			CN > MCI		
	*t*-stats		Effect-size	*t*-stats		Effect-size
	Vol (%)	<t>	g_**max**_	Vol (%)	<t>	g_**max**_
JHU Atlas						
Anterior thalamic radiation L	–	–	–	4.52	−2.347	−0.637
Anterior thalamic radiation R	–	–	–	2.31	−2.587	−0.690
Cingulum hippocampus L	–	–	–	5.57	−2.521	−0.642
Forceps major	2.70	2.583	1.057	–	–	–
Forceps minor	–	–	–	1.55	−2.276	−0.624
Inferior fronto-occipital fasciculus L	0.96	2.765	1.057	2.48	−2.500	−0.704
Inferior fronto-occipital fasciculus R	0.30	2.325	0.804	1.66	−2.526	−0.695
Inferior longitudinal fasciculus L	1.05	2.663	1.036	3.63	−2.506	−0.876
Inferior longitudinal fasciculus R	–	–	–	1.65	−2.572	−0.695
Superior longitudinal fasciculus L	–	–	–	3.06	−2.445	−0.778
Uncinate fasciculus L	–	–	–	0.75	−2.404	−0.632
Superior longitudinal fasciculus temporal L	–	–	–	5.62	−2.422	−0.712
ICBM81 Atlas						
Middle cerebellar peduncle	0.95	2.554	0.709	–	–	–
Anterior corona radiata R	–	–	–	3.90	−2.291	−0.624
Posterior thalamic radiation L	–	–	–	6.06	−2.599	−0.623
Sagittal stratum R	–	–	–	10.82	−2.590	−0.695
Cingulum (hippocampus) L	–	–	–	9.52	−2.521	−0.613
Fornix (cres) / Stria terminalis R	–	–	–	11.30	−3.017	−0.647
Fornix (cres) / Stria terminalis L	–	–	–	4.09	−2.486	−0.606
Superior longitudinal fasciculus L	–	–	–	5.22	−2.372	−0.778

Only significant clusters with large effect size (g > 0.61) are presented. Comprehensive results, including medium and lower effect sizes, can be found in Supplementary Tables S1, S2. Vol (%): Percentage of the cluster’s volume within the respective white matter area, <t>: Mean *t* value within the cluster; gmax: Maximum g value within the cluster.

Figure 1B shows the statistical voxel-based differences between groups for the fw-FA metric. Compared to the CN group, the MCI group exhibited extensive clusters characterized by lower fw-FA values, with a coefficient |g| > 0.61. These clusters were situated within the right sagittal stratum, left cingulum (hippocampus), and right fornix (crus)/stria terminalis. Table 2B presents the findings for clusters with |g| > 0.61. Complete results for fw-FA, including large, medium, and small effect sizes, are shown in Supplementary Table S2.

### 3.2 DKI results

**Figure 2:**
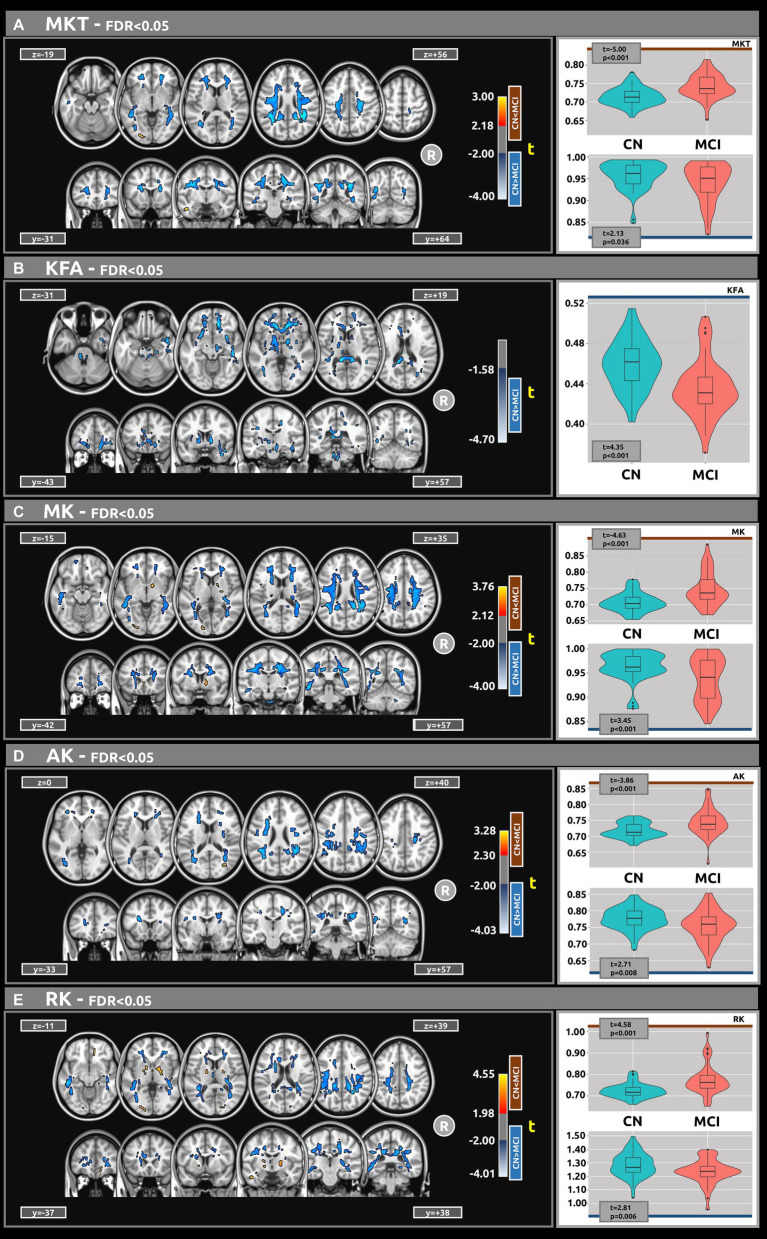
Voxel-based analyses of DKI metrics: **(A)** MKT, **(B)** KFA, **(C)** MK, **(D)** AK, and **(E)** Mk, respectively. *t*-value (at FDR < 0.05) are displayed in axial and coronal views. Group differences were identified for all metrics, typically showing large clusters, wherein the MCI group exhibited lower DKI metrics values compared to the CN group. Plots illustrate the mean diffusion values within the significant clusters.

Figure 2 displays the voxel-based differences between groups for DKI-related metrics. The MCI group generally exhibited reduced kurtosis values compared to the CN group. Significant differences were observed across various white matter regions, characterized by large clusters and |g| > 0.61. The white matter regions most associated with cognitive decline include the superior longitudinal fasciculus, superior fronto-occipital fasciculus, corpus callosum, and sagittal stratum. Smaller clusters indicating higher kurtosis values in MCI compared to CN (excluding KFA) were also identified.

Table 3 presents the outcomes for clusters with |g| > 0.61 for MKT and KFA metrics. Table 4 outlines the results for clusters with |g| > 0.61 for MK, AK, and RK metrics. Complete DKI results for large, medium, and small effect sizes are shown in Supplementary Tables S3–S7 for MKT, KFA, MK, AK, and RK, respectively.

**Table 3:** Results for DKI (**(A)** MKT and **(B)** KFA).

DKI (MKT & KFA)						
(A)	MKT					
	CN < MCI			CN > MCI		
	*t*-stats		Effect-size	*t*-stats		Effect-size
	Vol (%)	<t>	g_max_	Vol (%)	<t>	g_max_
JHU Atlas						
Cortical spinal tract R	0.24	2.552	0.697	–	–	–
Forceps major	0.97	2.623	0.640	–	–	–
Forceps minor	0.22	2.663	0.739	–	–	–
Inferior fronto-occipital fasciculus L	0.38	2.588	0.620	16.43	−2.220	−0.708
Inferior longitudinal fasciculus L	0.58	2.652	0.849	14.73	−2.347	−0.755
Superior longitudinal fasciculus L	–	–	–	18.91	−2.539	−0.747
Superior longitudinal fasciculus R	–	–	–	20.89	−2.542	−0.732
Uncinate fasciculus R	0.28	2.587	0.660	–	–	–
Superior longitudinal fasciculus temporal L	–	–	–	31.72	−2.556	−0.747
Superior longitudinal fasciculus temporal R	–	–	–	35.23	−2.574	−0.732
ICBM81 Atlas						
Splenium of corpus callosum	–	–	–	16.08	−2.435	−0.661
Posterior limb of internal capsule R	0.77	2.492	0.649	–	–	–
Posterior thalamic radiation L	–	–	–	28.23	−2.189	−0.697
Superior longitudinal fasciculus R	–	–	–	56.86	−2.564	−0.705
Superior longitudinal fasciculus L	–	–	–	52.94	−2.570	−0.637
Superior fronto-occipital fasciculus R	–	–	–	72.19	−2.654	−0.665
Superior fronto-occipital fasciculus L	–	–	–	74.36	−2.880	−0.649

(B)	KFA		
	CN > MCI		
	*t*-stats		Effect-size
	Vol (%)	<t>	g_max_
JHU Atlas			
Anterior thalamic radiation L	11.08	−2.069	−0.717
Anterior thalamic radiation R	14.61	−2.318	−0.934
Cortical spinal tract L	3.15	−2.169	−0.626
Cortical spinal tract R	7.69	−2.290	−0.630
Cingulum cingulate gyrus L	6.67	−2.137	−0.958
Cingulum cingulate gyrus R	2.25	−2.152	−0.649
Cingulum hippocampus R	9.48	−2.824	−0.845
Forceps major	6.69	−2.461	−0.690
Forceps minor	17.93	−2.411	−0.958
Inferior fronto-occipital fasciculus L	10.43	−2.123	−0.788
Inferior fronto-occipital fasciculus R	14.03	−2.312	−0.934
Inferior longitudinal fasciculus L	2.09	−2.315	−0.685
Inferior longitudinal fasciculus R	12.78	−2.311	−0.748
Superior longitudinal fasciculus L	3.41	−2.271	−0.685
Superior longitudinal fasciculus R	2.46	−2.269	−0.614
Uncinate fasciculus L	17.83	−2.125	−0.788
Uncinate fasciculus R	30.53	−2.425	−0.903
Superior longitudinal fasciculus temporal L	5.75	−2.250	−0.685
Superior longitudinal fasciculus temporal R	4.72	−2.343	−0.614
ICBM81 Atlas			
Genu of corpus callosum	33.34	−2.342	−0.898
Splenium of corpus callosum	21.39	−2.441	−0.709
Medial lemniscus R	30.14	−2.341	−0.664
Superior cerebellar peduncle R	15.83	−2.429	−0.639
Cerebral peduncle R	20.06	−2.352	−0.665
Cerebral peduncle L	5.62	−2.007	−0.628
Anterior limb of internal capsule R	13.96	−2.438	−0.647
Posterior limb of internal capsule R	7.88	−2.277	−0.630
Posterior limb of internal capsule L	20.34	−1.998	−0.656
Retrolenticular part of internal capsule L	11.46	−2.071	−0.642
Anterior corona radiata R	22.69	−2.607	−0.934
Anterior corona radiata L	13.65	−2.194	−0.780
Posterior thalamic radiation L	3.32	−2.322	−0.627
Sagittal stratum R	25.27	−2.399	−0.729
External capsule R	9.86	−2.192	−0.623
External capsule L	18.20	−2.162	−0.788
Cingulum (hippocampus) R	25.65	−2.738	−0.845
Fornix (cres) / Stria terminalis R	17.44	−3.071	−0.705

Only significant clusters with large effect size (*g* > 0.61) are presented. Comprehensive results, including medium and lower effect sizes, can be found in Supplementary Tables S3, S4. Vol (%): Percentage of the cluster’s volume within the respective white matter area, <t>: Mean *t* value within the cluster; gmax: Maximum g value within the cluster.

**Table 4:** Results for DKI (**(A)** MK, **(B)** AK, and **(C)** RK).

DKI (MK - AK - RK)						
(A)	MK					
	CN < MCI	CN > MCI				
	*t*-stats		Effect-size	*t*-stats		Effect-size
	Vol (%)	<t>	g_max_	Vol (%)	<t>	g_max_
JHU Atlas						
Anterior thalamic radiation R	1.38	2.508	0.708	–	–	–
Cortical spinal tract L	–	–	–	20.04	−2.306	−0.612
Cortical spinal tract R	0.54	2.588	0.774	–	–	–
Forceps major	0.90	2.660	0.641	4.28	−1.949	−0.617
Forceps minor	0.73	2.710	0.784	–	–	–
Inferior fronto-occipital fasciculus L	0.48	2.668	0.634	20.67	−2.224	−0.698
Inferior fronto-occipital fasciculus R	–	–	–	19.25	−2.203	−0.640
Inferior longitudinal fasciculus L	0.79	2.639	0.827	20.19	−2.347	−0.737
Superior longitudinal fasciculus L	–	–	–	20.04	−2.509	−0.737
Superior longitudinal fasciculus R	–	–	–	20.68	−2.523	−0.746
Uncinate fasciculus R	0.44	2.724	0.708	–	–	–
Superior longitudinal fasciculus temporal L	–	–	–	33.98	−2.533	−0.737
Superior longitudinal fasciculus temporal R	–	–	–	35.90	−2.581	−0.746
ICBM81 Atlas						
Posterior limb of internal capsule R	3.17	2.429	0.753	–	–	–
Retrolenticular part of internal capsule R	–	–	–	57.97	−2.293	−0.640
Superior corona radiata L	–	–	–	62.85	−2.257	−0.632
Posterior thalamic radiation R	–	–	–	50.28	−2.226	−0.683
Posterior thalamic radiation L	–	–	–	45.45	−2.135	−0.698
Sagittal stratum R	–	–	–	36.85	−2.146	−0.653
Sagittal stratum L	–	–	–	32.99	−2.390	−0.623
Superior longitudinal fasciculus R	–	–	–	57.21	−2.569	−0.724
Superior longitudinal fasciculus L	–	–	–	53.93	−2.558	−0.618

(B)	AK					
	CN < MCI			CN > MCI		
	*t*-stats		Effect-size	*t*-stats		Effect-size
	Vol (%)	<t>	g_max_	Vol (%)	<t>	g_max_
JHU Atlas						
Cortical spinal tract R	–	–	–	7.60	−2.270	−0.681
Inferior fronto-occipital fasciculus L	–	–	–	5.80	−2.253	−0.675
Inferior longitudinal fasciculus R	0.29	2.637	0.736	–	–	–
Superior longitudinal fasciculus L	–	–	–	10.56	−2.463	−0.846
Superior longitudinal fasciculus R	–	–	–	14.53	−2.521	−0.710
Superior longitudinal fasciculus temporal L	–	–	–	16.55	−2.475	−0.846
Superior longitudinal fasciculus temporal R	–	–	–	23.48	−2.680	−0.710
ICBM81 Atlas						
Superior corona radiata L	–	–	–	19.69	−2.277	−0.620
Superior longitudinal fasciculus R	–	–	–	37.67	−2.590	−0.686
Superior longitudinal fasciculus L	–	–	–	29.48	−2.425	−0.741

(C)	RK					
	CN < MCI			CN > MCI		
	*t*-stats		Effect-size	*t*-stats		Effect-size
	Vol (%)	<t>	g_max_	Vol (%)	<t>	g_max_
JHU Atlas						
Anterior thalamic radiation L	0.37	2.413	0.671	–	–	–
Anterior thalamic radiation R	0.93	2.753	0.934	–	–	–
Cortical spinal tract R	0.51	2.676	0.787	–	–	–
Forceps major	1.90	2.607	0.831	–	–	–
Forceps minor	0.57	2.864	0.827	–	–	–
Inferior fronto-occipital fasciculus L	0.83	2.751	0.839	13.70	−2.232	−0.705
Inferior fronto-occipital fasciculus R	–	–	–	13.19	−2.183	−0.809
Inferior longitudinal fasciculus L	1.30	2.657	0.828	17.14	−2.345	−0.783
Inferior longitudinal fasciculus R	–	–	–	11.31	−2.093	−0.606
Superior longitudinal fasciculus L	–	–	–	15.26	−2.266	−0.723
Superior longitudinal fasciculus R	–	–	–	10.01	−2.112	−0.641
Uncinate fasciculus R	0.74	2.719	0.730	–	–	–
Superior longitudinal fasciculus temporal L	–	–	–	26.37	−2.307	−0.723
Superior longitudinal fasciculus temporal R	–	–	–	19.73	−2.089	−0.622
ICBM81 Atlas						
Splenium of corpus callosum	–	–	–	23.97	−2.345	−0.627
Anterior limb of internal capsule R	1.21	2.471	0.651	–	–	–
Anterior limb of internal capsule L	1.19	2.481	0.665	–	–	–
Posterior limb of internal capsule R	6.79	2.395	0.734	–	–	–
Posterior limb of internal capsule L	3.49	2.291	0.637	–	–	–
Retrolenticular part of internal capsule R	–	–	–	59.13	−2.363	−0.809
Retrolenticular part of internal capsule L	–	–	–	16.20	−1.985	−0.642
Posterior corona radiata R	–	–	–	44.50	−2.401	−0.643
Posterior thalamic radiation R	–	–	–	32.80	−2.173	−0.661
Posterior thalamic radiation L	–	–	–	31.25	−2.146	−0.704
Sagittal stratum R	–	–	–	35.77	−1.999	−0.636
Sagittal stratum L	–	–	–	26.94	−2.348	−0.705
Superior longitudinal fasciculus L	–	–	–	37.21	−2.144	−0.649

Only significant clusters with large effect size (*g* > 0.61) are presented. Comprehensive results, including medium and lower effect sizes, can be found in Supplementary Tables S5–S7. Vol (%): Percentage of the cluster’s volume within the respective white matter area, <t>: Mean *t* value within the cluster; gmax: Maximum g value within the cluster.

### 3.3 MSDKI results

**Figure 3:**
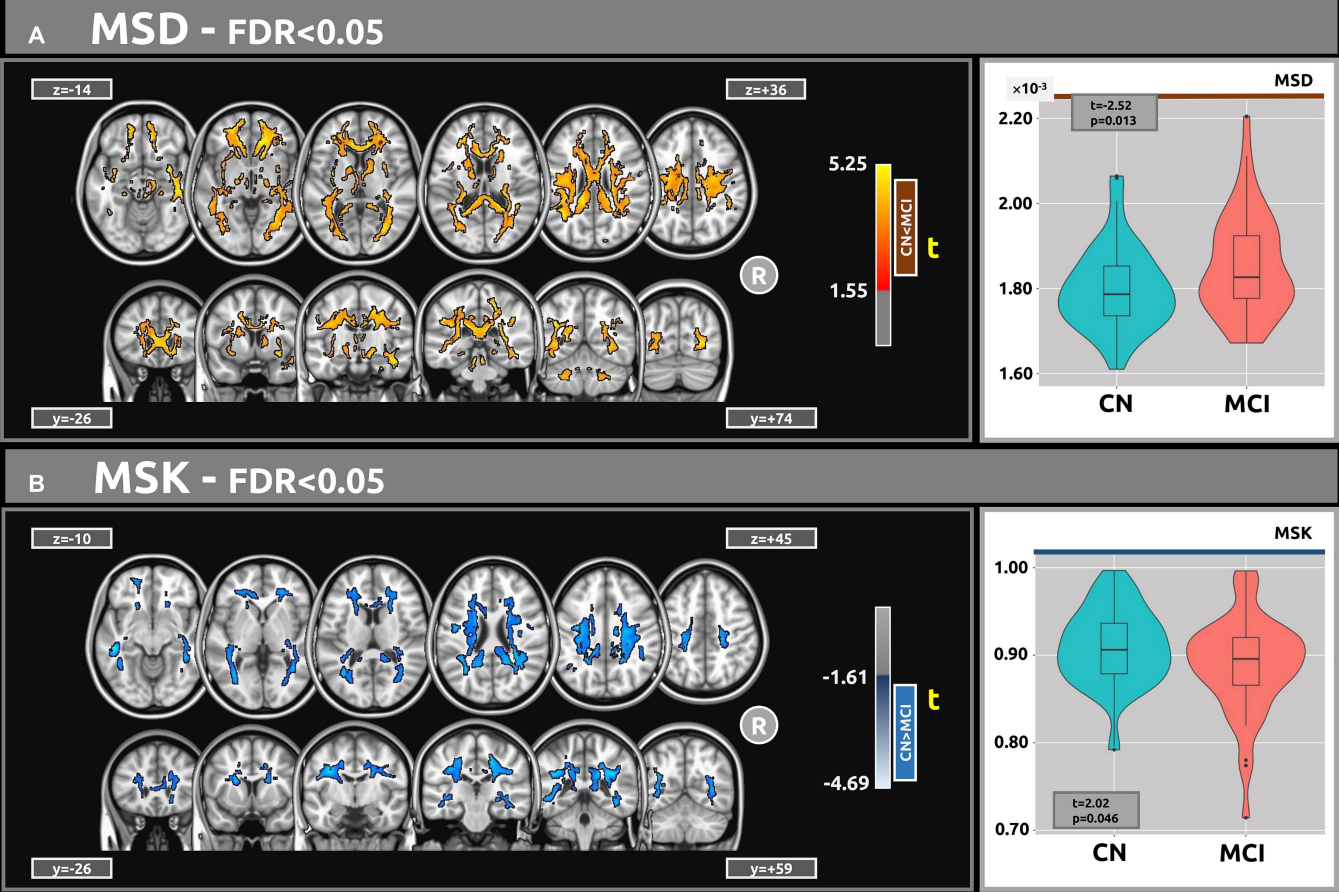
Voxel-based analyses of MSDKI metrics (t-value at FDR < 0.05) are presented in axial and coronal views. **(A)** In comparison with the CN group, MCI participants exhibited higher MSD values. **(B)** Contrasted with CN, the MCI group demonstrated lower MSK values. Plots show the mean diffusion values within the significant clusters.

Figure 3A shows the voxel-based statistical differences between groups regarding the MSD metric. The MCI group demonstrated higher water diffusion levels than the CN group. Extensive clusters with |g| > 0.61 were observed across various white matter regions. Notably, the MSD findings are aligned with those of the *f* index. Table 5A provides detailed information on the results for the significant clusters with |g| > 0.61. The complete results for the MSD, which include large, medium, and small effect sizes, are provided in Supplementary Table S8.

**Table 5:** Results for MSDKI (**(A)** MSD and **(B)** MSK).

MSDKI			
(A)	MSD		
	CN < MCI		
	t-stats		Effect-size
	Vol (%)	<t>	g_max_
JHU Atlas			
Anterior thalamic radiation L	28.87	2.176	0.777
Anterior thalamic radiation R	31.07	2.219	0.802
Cortical spinal tract R	32.29	2.359	0.698
Cingulum cingulate gyrus L	30.45	2.251	0.736
Cingulum cingulate gyrus R	20.61	2.222	0.671
Cingulum hippocampus R	24.34	2.794	0.814
Forceps major	28.48	2.389	0.701
Forceps minor	33.75	2.608	0.841
Inferior fronto-occipital fasciculus L	34.25	2.190	0.777
Inferior fronto-occipital fasciculus R	42.38	2.478	0.841
Inferior longitudinal fasciculus L	20.60	2.125	0.787
Inferior longitudinal fasciculus R	44.61	2.601	0.864
Superior longitudinal fasciculus L	27.09	2.321	0.820
Superior longitudinal fasciculus R	23.28	2.189	0.703
Uncinate fasciculus L	39.63	2.227	0.750
Uncinate fasciculus R	46.44	2.707	0.804
Superior longitudinal fasciculus temporal L	40.16	2.287	0.714
Superior longitudinal fasciculus temporal R	32.91	2.137	0.703
ICBM81 Atlas			
Genu of corpus callosum	56.28	2.779	0.781
Splenium of corpus callosum	70.59	2.640	0.620
Superior cerebellar peduncle R	25.81	2.330	0.671
Superior cerebellar peduncle L	26.81	2.325	0.668
Cerebral peduncle R	46.66	2.246	0.668
Anterior corona radiata R	51.86	2.412	0.841
Anterior corona radiata L	48.19	2.014	0.645
Superior corona radiata R	54.67	2.316	0.639
Superior corona radiata L	48.52	2.174	0.641
Sagittal stratum R	66.65	2.634	0.660
External capsule R	26.31	2.329	0.622
External capsule L	34.67	2.201	0.624
Cingulum (cingulate gyrus) L	36.46	2.181	0.736
Cingulum (hippocampus) R	47.49	2.900	0.814
Superior longitudinal fasciculus L	69.83	2.341	0.663
Uncinate fasciculus R	76.84	3.186	0.666

(B)	MSK		
	CN > MCI		
	*t*-stats		Effect-size
	Vol (%)	<t>	g_max_
JHU Atlas			
Inferior fronto-occipital fasciculus L	20.61	−2.040	−0.709
Inferior fronto-occipital fasciculus R	22.05	−2.099	−0.680
Inferior longitudinal fasciculus L	17.70	−2.173	−0.753
Inferior longitudinal fasciculus R	19.62	−2.095	−0.630
Superior longitudinal fasciculus L	20.02	−2.478	−0.700
Superior longitudinal fasciculus R	20.13	−2.396	−0.729
Superior longitudinal fasciculus temporal L	33.22	−2.498	−0.700
Superior longitudinal fasciculus temporal R	34.23	−2.415	−0.729
ICBM81 Atlas			
Posterior thalamic radiation L	37.83	−2.119	−0.709
Superior longitudinal fasciculus R	56.39	−2.408	−0.706
Superior longitudinal fasciculus L	54.88	−2.519	−0.651

Only significant clusters with large effect size (g > 0.61) are presented. Comprehensive results, including medium and lower effect sizes, can be found in Supplementary Tables S8, S9. Vol (%): Percentage of the cluster’s volume within the respective white matter area, <t>: Mean *t* value within the cluster; gmax: Maximum g value within the cluster.

Figure 3B shows the voxel-based differences between groups for the MSK metric. The MCI participants demonstrated lower MSK values than the CN group. Clusters with |g| > 0.61 were observed across various white matter regions. Table 5B provides detailed information on the results for the significant clusters with |g| > 0.61. The complete results for the MSK, which include large, medium, and small effect sizes, are provided in Supplementary Table S9.

### 3.4 Voxel-based correlations with MMSE

**Figure 4:**
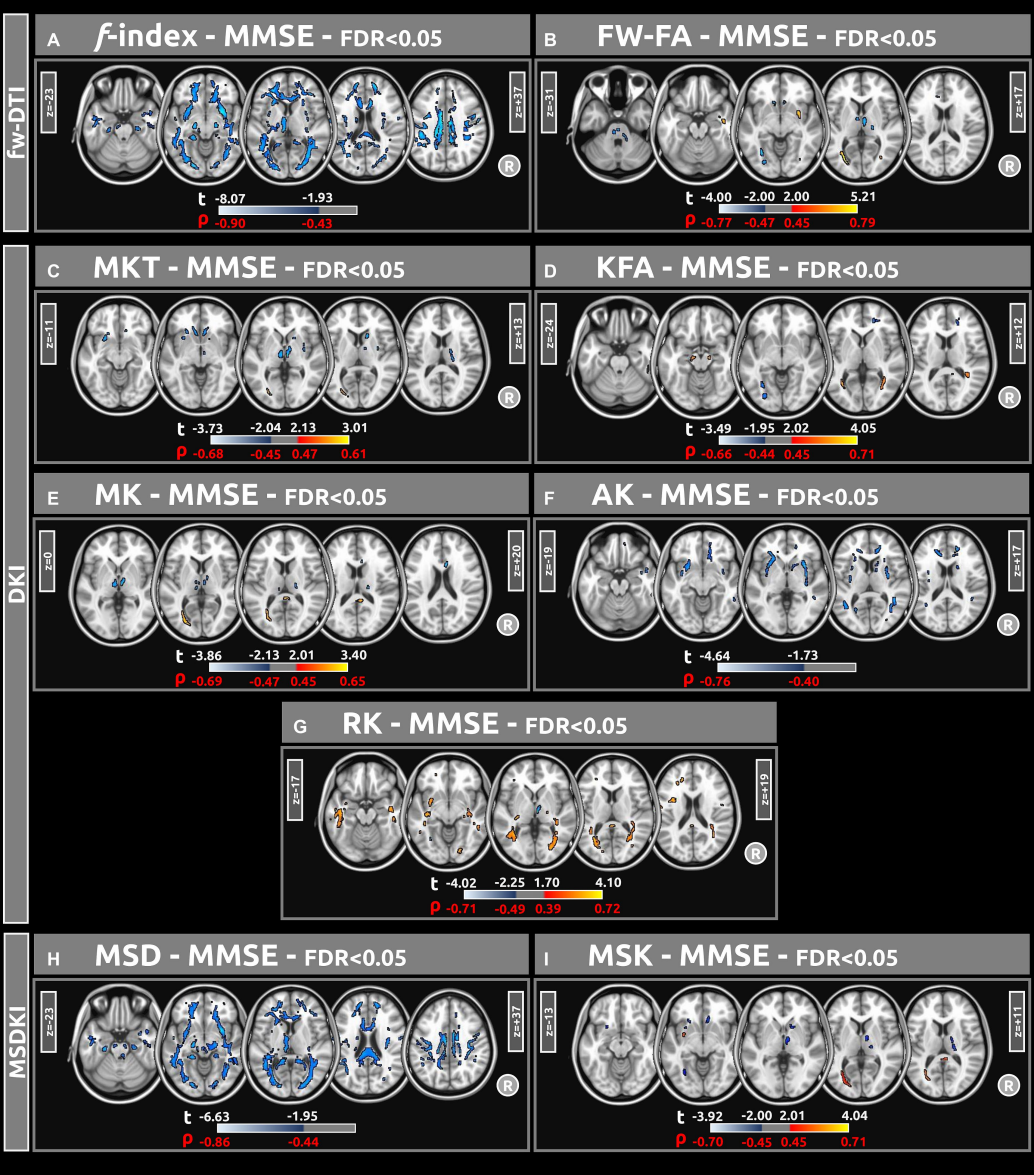
Voxel-based correlations between all diffusion metrics (**(A)** f index, **(B)** fw-FA, **(C)** MKT, **(D)** KFA, **(E)** MK, **(F)** AK, **(G)** RK, **(H)** MSD, and **(I)** MSK) and MMSE scores were computed using a linear model (at FDR < 0.05). Large clusters of correlations were found particularly for the f-index and MSD metrics. The parameters reported include *t*-values (t) and Spearman’s correlation coefficients (*ρ*).

Figure 4 displays voxel-based correlations between the diffusion metrics and MMSE scores. Notably, significant negative correlations were observed between MMSE scores and both the *f* index and MSD metric (with |ρ| > 0.44) across several comparable regions of white matter. Regarding other diffusion metrics, both positive and negative correlations with MMSE scores were identified. Detailed information on these correlations is provided in Tables 6–9.

**Table 6:** Voxel-based correlations between fw-DTI metrics (f-index and fw-FA) and MMSE scores at FDR > 0.05.

*f*-index					
	Negative Correlation				
	*t*-stats		Effect-size		
	Vol (%)	<t>	<ρ>	ρ_min_	ρ_max_
fw-DTI					
JHU Atlas					
Anterior thalamic radiation L	22.30	−2.970	−0.586	−0.437	−0.810
Anterior thalamic radiation R	5.76	−2.713	−0.552	−0.434	−0.821
Cortical spinal tract L	9.97	−2.807	−0.564	−0.437	−0.774
Cortical spinal tract R	6.68	−3.181	−0.610	−0.438	−0.783
Cingulum cingulate gyrus L	40.24	−3.369	−0.623	−0.439	−0.895
Cingulum cingulate gyrus R	25.90	−3.388	−0.631	−0.444	−0.846
Cingulum hippocampus L	27.12	−2.963	−0.587	−0.440	−0.742
Cingulum hippocampus R	17.57	−2.830	−0.570	−0.451	−0.707
Forceps major	17.34	−2.903	−0.579	−0.436	−0.791
Forceps minor	23.56	−2.844	−0.570	−0.438	−0.821
Inferior fronto-occipital fasciculus L	30.38	−2.869	−0.572	−0.437	−0.848
Inferior fronto-occipital fasciculus R	31.63	−2.944	−0.582	−0.434	−0.846
Inferior longitudinal fasciculus L	24.93	−2.744	−0.557	−0.436	−0.841
Inferior longitudinal fasciculus R	37.11	−2.956	−0.585	−0.439	−0.848
Superior longitudinal fasciculus L	19.28	−2.886	−0.575	−0.436	−0.806
Superior longitudinal fasciculus R	13.01	−2.929	−0.580	−0.439	−0.827
Uncinate fasciculus L	34.59	−2.997	−0.588	−0.438	−0.848
Uncinate fasciculus R	33.74	−3.206	−0.608	−0.434	−0.846
Superior longitudinal fasciculus temporal L	29.31	−2.855	−0.571	−0.436	−0.806
Superior longitudinal fasciculus temporal R	23.62	−2.910	−0.579	−0.439	−0.821
ICBM81 Atlas					
Genu of corpus callosum	38.07	−2.822	−0.567	−0.438	−0.776
Body of corpus callosum	37.33	−3.191	−0.608	−0.435	−0.852
Splenium of corpus callosum	25.85	−2.777	−0.562	−0.441	−0.828
Corticospinal tract R	0.66	−3.027	−0.600	−0.570	−0.629
Corticospinal tract L	2.92	−2.392	−0.509	−0.452	−0.599
Superior cerebellar peduncle L	5.44	−2.219	−0.484	−0.445	−0.558
Cerebral peduncle R	18.70	−3.626	−0.662	−0.555	−0.783
Cerebral peduncle L	28.58	−3.090	−0.599	−0.448	−0.774
Anterior limb of internal capsule L	11.17	−2.342	−0.502	−0.441	−0.653
Posterior limb of internal capsule L	11.57	−2.475	−0.522	−0.439	−0.688
Retrolenticular part of internal capsule R	9.18	−2.578	−0.536	−0.444	−0.673
Retrolenticular part of internal capsule L	18.71	−2.524	−0.529	−0.437	−0.682
Anterior corona radiata R	20.59	−2.737	−0.558	−0.434	−0.770
Anterior corona radiata L	36.16	−2.891	−0.573	−0.440	−0.848
Superior corona radiata R	11.55	−2.597	−0.537	−0.435	−0.738
Superior corona radiata L	5.98	−3.367	−0.623	−0.438	−0.780
Posterior corona radiata R	2.95	−2.891	−0.579	−0.454	−0.677
Posterior corona radiata L	6.49	−3.271	−0.602	−0.438	−0.819
Posterior thalamic radiation R	56.42	−2.952	−0.587	−0.446	−0.760
Posterior thalamic radiation L	40.57	−2.692	−0.552	−0.437	−0.695
Sagittal stratum R	41.29	−2.607	−0.541	−0.440	−0.700
Sagittal stratum L	8.20	−2.571	−0.536	−0.438	−0.675
External capsule R	35.04	−3.111	−0.598	−0.445	−0.842
External capsule L	31.02	−2.981	−0.587	−0.443	−0.810
Cingulum (cingulate gyrus) R	35.65	−3.600	−0.655	−0.450	−0.846
Cingulum (cingulate gyrus) L	55.58	−3.994	−0.684	−0.439	−0.895
Cingulum (hippocampus) R	37.46	−2.856	−0.574	−0.448	−0.707
Cingulum (hippocampus) L	46.84	−3.012	−0.594	−0.442	−0.737
Fornix (cres) / Stria terminalis R	2.49	−2.230	−0.485	−0.449	−0.563
Superior longitudinal fasciculus R	29.82	−2.981	−0.586	−0.446	−0.827
Superior longitudinal fasciculus L	38.29	−2.910	−0.579	−0.441	−0.774
Superior fronto-occipital fasciculus L	0.99	−2.286	−0.495	−0.490	−0.503
Uncinate fasciculus R	76.58	−4.230	−0.715	−0.477	−0.807
Uncinate fasciculus L	19.68	−2.417	−0.512	−0.441	−0.638
Tapetum R	9.90	−2.908	−0.580	−0.466	−0.698
Tapetum L	2.67	−2.524	−0.530	−0.464	−0.607

	Negative correlation					Positive correlation				
	*t*-stats		Effect-size			*t*-stats		Effect-size		
	Vol (%)	<t>	<ρ>	ρ_min_	ρ_max_	Vol (%)	<t>	<ρ>	ρ_min_	ρ_max_
fw-FA										
JHU Atlas										
Anterior thalamic radiation L	0.36	−2.745	−0.562	−0.504	−0.647	–	–	–	–	–
Anterior thalamic radiation R	1.48	−2.915	−0.581	−0.478	−0.730	–	–	–	–	–
Cortical spinal tract L	0.91	−2.520	−0.529	−0.451	−0.648	–	–	–	–	–
Cortical spinal tract R	1.12	−2.591	−0.540	−0.460	−0.658	–	–	–	–	–
Cingulum cingulate gyrus L	1.37	−2.558	−0.534	−0.468	−0.653	–	–	–	–	–
Forceps major	0.49	−2.787	−0.561	−0.457	−0.708	1.69	3.030	0.588	0.457	0.792
Forceps minor	0.29	−2.630	−0.544	−0.468	−0.661	–	–	–	–	–
Inferior fronto-occipital fasciculus L	1.92	−3.063	−0.595	−0.456	−0.774	1.02	3.144	0.600	0.456	0.792
Inferior fronto-occipital fasciculus R	–	–	–	–	–	1.84	2.579	0.537	0.453	0.667
Inferior longitudinal fasciculus L	2.27	−3.023	−0.590	−0.456	−0.774	0.99	3.152	0.600	0.456	0.792
Inferior longitudinal fasciculus R	–	–	–	–	–	2.87	2.440	0.518	0.450	0.640
Superior longitudinal fasciculus L	0.31	−2.560	−0.536	−0.481	−0.632	–	–	–	–	–
Uncinate fasciculus R	–	–	–	–	–	4.01	2.722	0.557	0.456	0.667
Superior longitudinal fasciculus temporal L	0.22	−2.407	−0.514	−0.481	−0.559	–	–	–	–	–
ICBM81 Atlas										
Middle cerebellar peduncle	0.62	−2.427	−0.515	−0.453	−0.601	–	–	–	–	–
Pontine crossing tract	10.93	−2.540	−0.531	−0.460	−0.658	–	–	–	–	–
Genu of corpus callosum	3.23	−2.772	−0.565	−0.470	−0.661	–	–	–	–	–
Corticospinal tract R	0.37	−2.293	−0.494	−0.467	−0.545	–	–	–	–	–
Corticospinal tract L	2.48	−2.312	−0.499	−0.451	−0.547	–	–	–	–	–
Inferior cerebellar peduncle R	1.03	−2.321	−0.499	−0.457	−0.551	–	–	–	–	–
Posterior limb of internal capsule R	0.75	−2.629	−0.547	−0.493	−0.593	–	–	–	–	–
Posterior thalamic radiation R	–	–	–	–	–	1.61	2.368	0.507	0.464	0.586
Posterior thalamic radiation L	0.75	−3.464	−0.634	−0.472	−0.774	5.71	2.949	0.578	0.456	0.792
Sagittal stratum R	–	–	–	–	–	3.05	2.571	0.537	0.453	0.640
Sagittal stratum L	0.22	−2.588	−0.540	−0.504	−0.616	–	–	–	–	–
External capsule R	–	–	–	–	–	4.95	2.742	0.560	0.458	0.667
Cingulum (cingulate gyrus) L	1.74	−2.665	−0.549	−0.469	−0.632	–	–	–	–	–
Uncinate fasciculus R	–	–	–	–	–	28.95	2.694	0.553	0.456	0.665

Large significant clusters with negative correlations were found for f-index. Vol (%): Percentage of the cluster’s volume within the respective white matter area; <t>: Mean *t*-value within the cluster. Effect sizes were determined using Spearman’s correlation coefficient (ρ), with a large effect size defined as *ρ* > 0.44. <ρ>: Mean Spearman’s correlation coefficient within the significant clusters. Ρmin and ρmax: Minimum and maximum values of the Spearman’s correlation coefficient within the significant clusters, respectively.

**Table 7:** Voxel-based correlations between DKI metrics (MKT and KFA) and MMSE scores at FDR > 0.05.

DKI (MKT & KFA)										
	Negative correlation					Positive correlation				
	*t*-stats		Effect-size			*t*-stats		Effect-size		
	Vol (%)	<t>	<ρ>	ρ_min_	ρ_max_	Vol (%)	<t>	<ρ>	ρ_min_	ρ_max_
MKT										
JHU Atlas										
Anterior thalamic radiation L	1.21	−2.566	−0.537	−0.476	−0.624	–	–	–	–	–
Anterior thalamic radiation R	2.50	−2.549	−0.533	−0.455	−0.681	–	–	–	–	–
Cortical spinal tract L	–	–	–	–	–	0.58	2.437	0.519	0.485	0.566
Forceps major	–	–	–	–	–	0.66	2.412	0.514	0.470	0.609
Forceps minor	0.43	−2.367	−0.508	−0.472	−0.569	–	–	–	–	–
Inferior fronto-occipital fasciculus L	0.90	−2.406	−0.513	−0.460	−0.601	0.59	2.414	0.514	0.470	0.609
Inferior longitudinal fasciculus L	–	–	–	–	–	0.64	2.415	0.515	0.472	0.609
Uncinate fasciculus L	2.86	−2.425	−0.516	−0.459	−0.601	–	–	–	–	–
ICBM81 Atlas										
Genu of corpus callosum	3.15	−2.424	−0.517	−0.472	−0.593	–	–	–	–	–
Anterior limb of internal capsule R	1.94	−2.295	−0.496	−0.463	−0.555	–	–	–	–	–
Posterior limb of internal capsule R	2.98	−2.516	−0.528	−0.458	−0.622	–	–	–	–	–
Anterior corona radiata L	0.72	−2.364	−0.507	−0.466	−0.565	–	–	–	–	–
External capsule R	1.94	−2.675	−0.553	−0.494	−0.627	–	–	–	–	–
External capsule L	4.55	−2.459	−0.521	−0.461	−0.601	–	–	–	–	–
KFA										
JHU Atlas										
Anterior thalamic radiation R	0.61	−2.178	−0.477	−0.438	−0.594	–	–	–	–	–
Cortical spinal tract L	–	–	–	–	–	1.51	2.610	0.542	0.470	0.643
Cortical spinal tract R	–	–	–	–	–	0.61	2.828	0.571	0.506	0.676
Forceps major	0.38	−2.595	−0.542	−0.458	−0.604	0.92	2.651	0.544	0.454	0.711
Forceps minor	0.95	−2.316	−0.498	−0.438	−0.635	–	–	–	–	–
Inferior fronto-occipital fasciculus L	1.47	−2.502	−0.527	−0.458	−0.658	0.77	2.814	0.567	0.472	0.711
Inferior fronto-occipital fasciculus R	0.24	−2.120	−0.467	−0.441	−0.505	1.39	2.384	0.509	0.450	0.628
Inferior longitudinal fasciculus L	1.58	−2.513	−0.529	−0.458	−0.658	0.82	2.804	0.565	0.472	0.711
Inferior longitudinal fasciculus R	–	–	–	–	–	1.28	2.399	0.511	0.454	0.628
Uncinate fasciculus R	0.49	−2.141	−0.471	−0.441	−0.510	–	–	–	–	–
Superior longitudinal fasciculus temporal R	–	–	–	–	–	0.32	2.185	0.479	0.456	0.519
ICBM81 Atlas										
Splenium of corpus callosum	0.81	−2.638	−0.548	−0.505	−0.604	–	–	–	–	–
Corticospinal tract L	–	–	–	–	–	0.29	2.374	0.509	0.490	0.524
Cerebral peduncle R	–	–	–	–	–	4.74	2.828	0.571	0.506	0.676
Cerebral peduncle L	–	–	–	–	–	8.34	2.695	0.554	0.491	0.643
Anterior corona radiata R	2.01	−2.314	−0.497	−0.439	−0.634	–	–	–	–	–
Posterior thalamic radiation R	–	–	–	–	–	11.20	2.383	0.509	0.450	0.628
Posterior thalamic radiation L	0.30	−2.464	−0.521	−0.493	−0.544	5.30	2.791	0.564	0.475	0.711
Superior longitudinal fasciculus L	–	–	–	–	–	0.47	2.300	0.498	0.474	0.538

Vol (%): Percentage of the cluster’s volume within the respective white matter area; <t>: Mean *t*-value within the cluster. Effect sizes were determined using Spearman’s correlation coefficient (ρ), with a large effect size defined as *ρ* > 0.44. <ρ>: Mean Spearman’s correlation coefficient within the significant clusters. Ρmin and ρmax: Minimum and maximum values of the Spearman’s correlation coefficient within the significant clusters, respectively.

**Table 8:** Voxel-based correlations between DKI metrics (MK, AK, and RK) and MMSE scores at FDR > 0.05.

DKI (MK - AK - RK)										
	Negative correlation					Positive correlation				
	*t*-stats		Effect-size			*t*-stats		Effect-size		
	Vol (%)	<t>	<ρ>	ρ_min_	ρ_max_	Vol (%)	<t>	<ρ>	ρ_min_	ρ_max_
MK										
JHU Atlas										
Anterior thalamic radiation L	1.17	−2.438	−0.519	−0.470	−0.603	–	–	–	–	–
Anterior thalamic radiation R	1.24	−2.753	−0.560	−0.469	−0.693	–	–	–	–	–
Cortical spinal tract L	–	–	–	–	–	1.10	2.651	0.548	0.471	0.647
Forceps major	–	–	–	–	–	2.39	2.511	0.528	0.450	0.638
Inferior fronto-occipital fasciculus L	–	–	–	–	–	1.43	2.510	0.528	0.452	0.638
Inferior longitudinal fasciculus L	–	–	–	–	–	1.42	2.528	0.530	0.452	0.638
ICBM81 Atlas										
Genu of corpus callosum	0.47	−2.676	−0.552	−0.495	−0.623	–	–	–	–	–
Body of corpus callosum	0.70	−2.911	−0.581	−0.501	−0.681	–	–	–	–	–
Splenium of corpus callosum	–	–	–	–	–	1.59	2.569	0.538	0.487	0.617
Posterior limb of internal capsule R	1.12	−2.459	−0.520	−0.473	−0.611	–	–	–	–	–
Posterior thalamic radiation L	–	–	–	–	–	5.25	2.403	0.512	0.452	0.588
AK										
JHU Atlas										
Anterior thalamic radiation L	0.99	−2.422	−0.513	−0.419	−0.660					
Anterior thalamic radiation R	2.24	−2.001	−0.446	−0.398	−0.560					
Cingulum cingulate gyrus L	4.03	−2.426	−0.513	−0.437	−0.687					
Cingulum cingulate gyrus R	1.85	−2.640	−0.540	−0.401	−0.734					
Forceps major	0.40	−2.720	−0.559	−0.497	−0.642					
Forceps minor	4.79	−2.320	−0.496	−0.398	−0.745					
Inferior fronto-occipital fasciculus L	5.28	−2.582	−0.536	−0.419	−0.722					
Inferior fronto-occipital fasciculus R	3.76	−2.430	−0.511	−0.400	−0.757					
Inferior longitudinal fasciculus L	0.56	−2.435	−0.518	−0.463	−0.596					
Inferior longitudinal fasciculus R	1.99	−2.330	−0.498	−0.401	−0.678					
Superior longitudinal fasciculus L	1.36	−2.321	−0.499	−0.415	−0.642					
Superior longitudinal fasciculus R	1.98	−2.555	−0.532	−0.404	−0.719					
Uncinate fasciculus L	13.17	−2.590	−0.538	−0.419	−0.722					
Uncinate fasciculus R	9.30	−2.321	−0.494	−0.399	−0.757					
Superior longitudinal fasciculus temporal L	1.37	−2.372	−0.507	−0.420	−0.597					
Superior longitudinal fasciculus temporal R	4.31	−2.497	−0.524	−0.404	−0.643					
ICBM81 Atlas										
Genu of corpus callosum	3.14	−2.282	−0.491	−0.401	−0.677					
Splenium of corpus callosum	0.53	−2.820	−0.573	−0.503	−0.642					
Anterior limb of internal capsule R	5.77	−1.990	−0.444	−0.401	−0.531					
Anterior limb of internal capsule L	1.13	−2.326	−0.499	−0.431	−0.580					
Retrolenticular part of internal capsule R	0.36	−1.894	−0.427	−0.408	−0.456					
Anterior corona radiata R	5.99	−2.328	−0.497	−0.398	−0.706					
Anterior corona radiata L	9.18	−2.500	−0.525	−0.419	−0.660					
Superior corona radiata L	1.74	−2.157	−0.472	−0.418	−0.553					
Posterior thalamic radiation R	1.03	−2.164	−0.472	−0.419	−0.600					
Posterior thalamic radiation L	0.40	−2.435	−0.517	−0.481	−0.561					
External capsule R	25.15	−2.603	−0.536	−0.403	−0.756					
External capsule L	23.66	−2.512	−0.526	−0.414	−0.722					
Cingulum (cingulate gyrus) L	0.36	−2.157	−0.473	−0.453	−0.497					
Superior longitudinal fasciculus R	4.34	−2.531	−0.529	−0.405	−0.643					
Superior longitudinal fasciculus L	2.39	−2.303	−0.497	−0.420	−0.572					
Uncinate fasciculus R	12.63	−2.297	−0.493	−0.414	−0.594					
Uncinate fasciculus L	0.53	−1.925	−0.430	−0.429	−0.431					
RK										
JHU Atlas										
Anterior thalamic radiation L	–	–	–	–	–	1.12	2.528	0.529	0.411	0.674
Anterior thalamic radiation R	0.72	−2.812	−0.570	−0.489	−0.675	–	–	–	–	–
Cortical spinal tract L	–	–	–	–	–	1.08	2.819	0.571	0.479	0.700
Cortical spinal tract R	–	–	–	–	–	2.08	2.008	0.447	0.392	0.547
Cingulum cingulate gyrus L	–	–	–	–	–	0.70	2.556	0.536	0.482	0.609
Forceps major	–	–	–	–	–	4.77	2.522	0.524	0.394	0.715
Forceps minor	–	–	–	–	–	0.32	2.524	0.531	0.464	0.609
Inferior fronto-occipital fasciculus L	0.37	−3.134	−0.609	−0.505	−0.708	8.52	2.439	0.514	0.404	0.715
Inferior fronto-occipital fasciculus R	–	–	–	–	–	7.36	2.137	0.468	0.391	0.626
Inferior longitudinal fasciculus L	0.45	−3.116	−0.607	−0.503	−0.708	8.83	2.382	0.505	0.405	0.715
Inferior longitudinal fasciculus R	–	–	–	–	–	12.77	2.212	0.480	0.391	0.656
Superior longitudinal fasciculus L	–	–	–	–	–	3.40	2.344	0.502	0.405	0.671
Superior longitudinal fasciculus R	–	–	–	–	–	5.33	2.059	0.455	0.391	0.686
Uncinate fasciculus L	–	–	–	–	–	3.97	2.388	0.509	0.404	0.674
Uncinate fasciculus R	–	–	–	–	–	1.33	2.227	0.482	0.399	0.616
Superior longitudinal fasciculus temporal L	–	–	–	–	–	5.85	2.272	0.491	0.405	0.638
Superior longitudinal fasciculus temporal R	–	–	–	–	–	12.19	2.082	0.459	0.391	0.686
ICBM81 Atlas										
Splenium of corpus callosum	–	–	–	–	–	2.84	2.548	0.534	0.416	0.628
Posterior limb of internal capsule R	–	–	–	–	–	1.20	1.872	0.423	0.396	0.481
Retrolenticular part of internal capsule R	–	–	–	–	–	24.93	2.174	0.475	0.391	0.608
Retrolenticular part of internal capsule L	–	–	–	–	–	4.01	2.230	0.484	0.418	0.562
Anterior corona radiata L	–	–	–	–	–	0.79	2.287	0.495	0.456	0.565
Posterior corona radiata R	–	–	–	–	–	0.97	1.961	0.439	0.396	0.510
Posterior thalamic radiation R	–	–	–	–	–	22.66	2.018	0.449	0.391	0.566
Posterior thalamic radiation L	–	–	–	–	–	25.64	2.497	0.521	0.409	0.715
Sagittal stratum R	–	–	–	–	–	15.62	2.101	0.463	0.391	0.586
Sagittal stratum L	–	–	–	–	–	22.59	2.385	0.508	0.408	0.631
External capsule R	–	–	–	–	–	1.05	1.933	0.434	0.396	0.505
External capsule L	–	–	–	–	–	4.24	2.318	0.499	0.404	0.641
Fornix (cres) / Stria terminalis R	–	–	–	–	–	6.05	1.996	0.445	0.397	0.548
Fornix (cres) / Stria terminalis L	–	–	–	–	–	3.02	2.119	0.466	0.407	0.520
Superior longitudinal fasciculus R	–	–	–	–	–	16.30	2.042	0.452	0.391	0.686
Superior longitudinal fasciculus L	–	–	–	–	–	4.68	2.518	0.529	0.408	0.643
Uncinate fasciculus L	–	–	–	–	–	17.82	2.139	0.469	0.410	0.580

Vol (%): Percentage of the cluster’s volume within the respective white matter area; <t>: Mean *t*-value within the cluster. Effect sizes were determined using Spearman’s correlation coefficient (ρ), with a large effect size defined as *ρ* > 0.44. <ρ>: Mean Spearman’s correlation coefficient within the significant clusters. Ρmin and ρmax: Minimum and maximum values of the Spearman’s correlation coefficient within the significant clusters, respectively.

**Table 9:** Voxel-based correlations between MSDKI metrics and MMSE scores at FDR > 0.05.

MSDKI					
MSD					
	Negative correlation				
	*t*-stats		Effect-size		
	Vol (%)	<t>	<ρ>	ρ_min_	ρ_max_
JHU Atlas					
Anterior thalamic radiation L	17.30	−2.872	−0.575	−0.440	−0.804
Anterior thalamic radiation R	6.77	−2.594	−0.537	−0.442	−0.758
Cortical spinal tract L	9.04	−2.771	−0.560	−0.440	−0.752
Cortical spinal tract R	9.18	−2.970	−0.587	−0.448	−0.758
Cingulum cingulate gyrus L	36.73	−3.372	−0.626	−0.444	−0.851
Cingulum cingulate gyrus R	25.93	−3.339	−0.628	−0.447	−0.841
Cingulum hippocampus L	32.53	−3.388	−0.635	−0.452	−0.790
Cingulum hippocampus R	23.23	−3.091	−0.601	−0.451	−0.776
Forceps major	22.76	−3.039	−0.595	−0.440	−0.825
Forceps minor	17.45	−2.757	−0.560	−0.439	−0.791
Inferior fronto-occipital fasciculus L	26.66	−2.865	−0.573	−0.440	−0.825
Inferior fronto-occipital fasciculus R	26.55	−2.811	−0.566	−0.442	−0.844
Inferior longitudinal fasciculus L	25.02	−2.812	−0.566	−0.440	−0.827
Inferior longitudinal fasciculus R	34.13	−2.823	−0.569	−0.442	−0.800
Superior longitudinal fasciculus L	15.18	−2.868	−0.575	−0.440	−0.793
Superior longitudinal fasciculus R	9.76	−2.913	−0.581	−0.441	−0.781
Uncinate fasciculus L	23.51	−2.853	−0.572	−0.443	−0.797
Uncinate fasciculus R	25.94	−3.144	−0.598	−0.442	−0.844
Superior longitudinal fasciculus temporal L	23.44	−2.845	−0.572	−0.440	−0.793
Superior longitudinal fasciculus temporal R	18.04	−2.864	−0.576	−0.441	−0.757
ICBM81 Atlas					
Genu of corpus callosum	31.42	−2.596	−0.540	−0.440	−0.723
Body of corpus callosum	39.11	−3.151	−0.606	−0.440	−0.821
Splenium of corpus callosum	45.92	−2.885	−0.578	−0.440	−0.774
Corticospinal tract R	2.94	−2.557	−0.534	−0.453	−0.629
Corticospinal tract L	4.53	−2.386	−0.510	−0.449	−0.577
Medial lemniscus R	0.43	−2.251	−0.489	−0.482	−0.503
Superior cerebellar peduncle R	9.88	−2.560	−0.536	−0.450	−0.597
Superior cerebellar peduncle L	9.58	−2.509	−0.528	−0.450	−0.590
Cerebral peduncle R	44.91	−2.886	−0.577	−0.448	−0.723
Cerebral peduncle L	30.60	−2.925	−0.580	−0.445	−0.752
Anterior limb of internal capsule L	4.97	−2.243	−0.487	−0.440	−0.609
Posterior limb of internal capsule R	0.59	−2.423	−0.514	−0.464	−0.563
Posterior limb of internal capsule L	6.66	−2.321	−0.499	−0.440	−0.647
Retrolenticular part of internal capsule R	11.57	−2.472	−0.521	−0.445	−0.639
Retrolenticular part of internal capsule L	19.97	−2.712	−0.555	−0.442	−0.699
Anterior corona radiata R	12.02	−2.545	−0.532	−0.443	−0.714
Anterior corona radiata L	15.12	−2.801	−0.563	−0.443	−0.794
Superior corona radiata R	4.33	−2.791	−0.565	−0.450	−0.686
Superior corona radiata L	3.09	−3.548	−0.651	−0.462	−0.755
Posterior corona radiata R	2.17	−2.849	−0.574	−0.457	−0.660
Posterior corona radiata L	4.74	−3.319	−0.616	−0.456	−0.790
Posterior thalamic radiation R	47.83	−2.765	−0.563	−0.444	−0.764
Posterior thalamic radiation L	39.97	−2.844	−0.572	−0.442	−0.731
Sagittal stratum R	38.38	−2.508	−0.527	−0.442	−0.716
Sagittal stratum L	10.80	−2.666	−0.549	−0.447	−0.713
External capsule R	27.36	−3.018	−0.586	−0.445	−0.844
External capsule L	19.24	−2.781	−0.563	−0.444	−0.771
Cingulum (cingulate gyrus) R	38.43	−3.519	−0.647	−0.447	−0.841
Cingulum (cingulate gyrus) L	57.00	−3.975	−0.688	−0.448	−0.851
Cingulum (hippocampus) R	52.18	−3.127	−0.605	−0.456	−0.776
Cingulum (hippocampus) L	56.71	−3.536	−0.651	−0.450	−0.790
Fornix (cres) / Stria terminalis R	0.89	−2.240	−0.486	−0.446	−0.533
Fornix (cres) / Stria terminalis L	9.16	−2.781	−0.565	−0.456	−0.705
Superior longitudinal fasciculus R	19.78	−2.923	−0.583	−0.447	−0.750
Superior longitudinal fasciculus L	21.26	−3.003	−0.593	−0.442	−0.738
Superior fronto-occipital fasciculus L	0.59	−2.232	−0.486	−0.473	−0.503
Uncinate fasciculus R	76.84	−4.258	−0.720	−0.490	−0.810
Uncinate fasciculus L	39.36	−2.824	−0.574	−0.472	−0.640
Tapetum R	8.56	−2.652	−0.549	−0.457	−0.630
Tapetum L	1.17	−2.601	−0.542	−0.500	−0.587

MSK										
	Negative correlation					Positive correlation				
	*t*-stats		Effect-size			*t*-stats		Effect-size		
	Vol (%)	<t>	<ρ>	ρ_min_	ρ_max_	Vol (%)	<t>	<ρ>	ρ_min_	ρ_max_
JHU Atlas										
Anterior thalamic radiation R	2.03	−2.484	−0.524	−0.449	−0.627	–	–	–	–	–
Cortical spinal tract L	–	–	–	–	–	1.19	2.677	0.553	0.468	0.616
Cortical spinal tract R	0.27	−2.137	−0.471	−0.450	−0.493	–	–	–	–	–
Forceps major	–	–	–	–	–	2.28	2.659	0.547	0.447	0.711
Inferior fronto-occipital fasciculus L	0.80	−2.669	−0.549	−0.471	−0.700	1.96	2.698	0.552	0.448	0.711
Inferior longitudinal fasciculus L	0.44	−2.941	−0.586	−0.499	−0.700	1.60	2.752	0.559	0.448	0.711
Uncinate fasciculus L	1.61	−2.488	−0.525	−0.471	−0.614	0.83	2.548	0.536	0.491	0.593
ICBM81 Atlas										
Genu of corpus callosum	0.84	−2.361	−0.507	−0.470	−0.560	–	–	–	–	–
Splenium of corpus callosum	–	–	–	–	–	0.79	2.481	0.525	0.491	0.592
Anterior limb of internal capsule R	2.77	−2.388	−0.511	−0.454	−0.581	–	–	–	–	–
Posterior limb of internal capsule R	4.48	−2.500	−0.525	−0.450	−0.637	–	–	–	–	–
Anterior corona radiata L	0.38	−2.313	−0.500	−0.474	−0.536	–	–	–	–	–
Posterior thalamic radiation L	–	–	–	–	–	6.86	2.704	0.552	0.448	0.711
External capsule L	2.54	−2.551	−0.534	−0.474	−0.614	1.81	2.555	0.537	0.491	0.593

Large significant clusters with negative correlations were found for MSD. Vol (%): Percentage of the cluster’s volume within the respective white matter area; <t>: Mean *t*-value within the cluster. Effect sizes were determined using Spearman’s correlation coefficient (ρ), with a large effect size defined as *ρ* > 0.44. <*ρ*>: Mean Spearman’s correlation coefficient within the significant clusters. Ρmin and ρmax: Minimum and maximum values of the Spearman’s correlation coefficient within the significant clusters, respectively.

## 4 Discussion

This study presents a comprehensive investigation of the relationship between diffusion metrics and cognitive status in CN and MCI cohorts. Through a multi-modal approach encompassing FWI, DKI, and MSDKI, this research offers valuable insights into the neurobiological mechanisms underpinning cognitive decline.

Our primary objective was to explore the complementary insights offered by FW-DTI and kurtosis MRI. Prior research has neither applied FW-DTI and DKI imaging to MCI, nor utilized MSDKI in this context. This multi-modal approach offers a comprehensive analysis of white matter alterations, providing insights that single-technique studies may overlook. Addressing the extracellular free-water component and capturing non-Gaussian diffusion behavior can lead to a precise characterization of tissue microstructure heterogeneity in MCI. The methodology is strengthened by the use of multi-shell data for free-water imaging. Multi-shell data allows for accurate separation of the free-water and tissue compartments, leading to reliable microstructural measurements. In contrast, single-shell approaches can result in biased estimates due to their limitations in disentangling these components. As noted by Golub et al., multiple b-values enhance the robustness and accuracy of FWI-DTI model (Golub et al., 2021).

FW-DTI accounts for the extracellular free-water component, providing more accurate tissue microstructure metrics, while kurtosis MRI captures non-Gaussian diffusion behavior, revealing tissue complexity not accessible with conventional DTI. Other advanced dMRI techniques, such as Neurite Orientation Dispersion and Density Imaging (NODDI) (Zhang et al., 2012) or intravoxel incoherent motion (IVIM) (Le Bihan, 2019), could offer additional insights in the context of cognitive decline. Previous studies in MCI and AD have shown that NODDI biomarkers of microstructure decrease, while those related to isotropic water diffusion increase (Fu et al., 2020). Additionally, IVIM may reveal early changes in microstructure and pseudoperfusion in MCI and AD cohorts (Bergamino et al., 2020, 2022). This study aimed to comprehensively assess the microstructural properties of MCI by utilizing both FW-DTI and DKI imaging techniques, and future studies could investigate a wider range of dMRI techniques in the context of cognitive decline.

A multi-shell dMRI acquisition computed the FWI-related metric, employing the algorithm developed by Hoy et al. (2014). This approach surpasses the constraints associated with single-shell acquisition algorithms (Pasternak et al., 2009). In analyzing the *f* index metric related to the free water content within brain tissue, pronounced differences between the CN and MCI groups were observed. Specifically, individuals with MCI demonstrated elevated free water levels across several white matter regions, potentially indicative of neuroinflammatory responses or axonal degradation processes (Dumont et al., 2019; Nakaya et al., 2022). These findings match those of prior studies showing higher free water measures in MCI and AD patients compared to CN controls (Dumont et al., 2019; Bergamino et al., 2021). Additionally, the identification of extensive clusters with large effect sizes, particularly in the corpus callosum, right cerebral peduncle, right sagittal stratum, and right uncinate fasciculus, highlights the vulnerability of these areas in the MCI cohort and suggests potential pathways for the spread of pathological changes.

Analysis of fw-FA metrics revealed a distinct pattern of white matter integrity degradation in the MCI group. Compared to the CN cohort, MCI subjects exhibited significantly lower fw-FA values in regions critical for cognitive processing and memory, including the right sagittal stratum, left cingulum (hippocampus), and right fornix (crus)/stria terminalis. Extensive clusters with a coefficient |g| > 0.61 in these areas point to compromised axonal integrity and myelination, likely reflecting early neurodegenerative changes. White matter clusters with elevated fw-FA values were also identified within the MCI group. These clusters had a large effect size, but their volumes were notably small.

The DKI model can overcome some of the limitations of DTI and has shown promise in differentiating between individuals with AD, MCI, and cognitively normal individuals (Struyfs et al., 2015; Chu et al., 2022; Xu et al., 2023). The lower kurtosis values across several white matter regions in the MCI group suggest a potential disruption in the microstructural integrity of these areas compared to the CN group. These significant differences, characterized by large clusters and large effect sizes, underscore the sensitivity of DKI-related metrics in detecting subtle variations in white matter microstructure. These findings align with previous research that demonstrated the utility of DKI in identifying microstructural changes associated with neurodegenerative diseases (Struyfs et al., 2015). In this study, the white matter regions most associated with cognitive decline are the superior longitudinal fasciculus, superior fronto-occipital fasciculus, corpus callosum, and sagittal stratum.

Smaller clusters with higher kurtosis values in the MCI group introduce an intriguing aspect of white matter pathology in MCI. This finding might suggest a heterogeneous response of white matter to the disease process, with specific areas possibly undergoing compensatory changes or differing in their susceptibility to neurodegeneration. While the volumes of these clusters were notably smaller, it should be noted that Dong et al. demonstrated non-monotonic behavior of DKI metrics in AD pathology, where subjects with milder amyloid deposition exhibited higher kurtosis than controls. This suggests that higher kurtosis values can be present in certain stages of disease progression or under specific pathological conditions (Dong et al., 2020). Additionally, several animal studies have shown an increase in kurtosis in the context of neuroinflammation, possibly reflecting processes such as astrogliosis and macrophage aggregation. These findings indicate that elevated kurtosis may be associated with early or specific pathological changes, rather than simply indicating a general decline in tissue integrity (Guan et al., 2021).

This study employed a novel approach utilizing the MSDKI model to investigate MCI and dementia. The findings provide evidence regarding the utility of MSD and MSK metrics in distinguishing between MCI and CN groups. The voxel-based statistical analysis revealed significant differences between MCI and CN groups, with MCI participants showing higher levels of water diffusion (MSD) and lower MSK values than the CN group. These differences were particularly pronounced in the white matter regions where clusters exhibited large effect sizes.

The increased free water diffusion observed in the MCI group may reflect microstructural degradation within white matter pathways. This degradation is likely a result of neurodegenerative processes that compromise the integrity of white matter, facilitating greater water movement. The alignment of MSD findings with the *f* index further corroborates this interpretation, suggesting a consistent pattern of white matter compromise by higher free water levels in individuals with MCI.

Conversely, the lower MSK values observed in the MCI group might indicate a reduction in the complexity of the microstructural environment. Kurtosis measures the deviation of water diffusion from a Gaussian distribution, with lower values suggesting a more homogeneous diffusion environment. This might be interpreted as a loss of microstructural complexity, potentially due to the simplification of neural pathways or a decrease in neuronal density, which are hallmark features of cognitive decline and neurodegeneration (Chu et al., 2022).

As assessed by MMSE scores, the voxel-based correlations in Figure 4 highlight the relationship between diffusion metrics and cognitive function. Significant negative correlations between MMSE scores and both the *f* index and the MSD metric across several white matter regions underscore the potential of these diffusion metrics as biomarkers for cognitive impairment. These correlations suggest that as the integrity of white matter decreases, cognitive function, measured by the MMSE, diminishes. This is consistent with previous research indicating that white matter integrity is crucial for efficient cognitive functioning, and white matter degradation can be linked to various neurodegenerative diseases and cognitive decline (Madden et al., 2009; Chen et al., 2023).

The analysis also revealed both positive and negative correlations between MMSE scores and other diffusion metrics across different brain regions. This indicates that certain brain regions may compensate for white matter degradation in others, or that changes in brain structure differentially impact different aspects of cognitive function.

Together, these findings revealed differences between groups across several white matter regions, including the forceps minor, inferior/superior longitudinal fasciculus, and corpus callosum. These areas are potentially associated with MCI or dementia. Therefore, studying the relationship between MCI and white matter microstructure using different dMRI methods may enhance our understanding and yield more comprehensive results. Although this study cannot definitively determine which metric or region is most sensitive to the earliest microstructural changes, the multi-modal approach aims to provide a comprehensive view of white matter alterations in MCI. We hypothesize that combining these metrics will enhance our ability to detect and characterize early neurodegenerative changes.

While this study employed multi-shell dMRI data and advanced dMRI models to address certain shortcomings of standard DTI, it remains subject to other limitations. Although DKI metrics have been reported to be less affected by the partial volume effect (PVE) than DTI metrics (Yang et al., 2013), they might still be influenced by PVEs. Another limitation is related to the MSDKI model, a variation of DKI that simplifies the complex metrics of DKI into a single mean signal metric. By focusing on a mean signal approach, MSDKI might overlook specific microstructural complexities that can be captured through the full DKI model. This simplification may lead to a loss of valuable information about the directionality of diffusion and microstructural heterogeneity within tissues.

In conclusion, this study highlights the complex link between diffusion metrics and cognitive status in cohorts of CN and MCI participants. It supplements previous research by revealing that microstructural changes, which are detectable through advanced diffusion imaging, may serve as early indicators of cognitive impairment. Additionally, multimodal diffusion MRI techniques provide new insights into the neurobiological mechanisms of cognitive decline while underscoring the value of advanced imaging for early detection.

## Data availability statement

Publicly available datasets were analyzed in this study. This data can be found at: https://adni.loni.usc.edu/.

## Ethics statement

The studies involving humans were approved by Alzheimer’s Disease Neuroimaging Initiative (ADNI) (National Institutes of Health Grant U01 AG024904) and DOD ADNI (Department of Defense award number W81XWH-12-2-0012) - https://adni.loni.usc.edu/. The studies were conducted in accordance with the local legislation and institutional requirements. The participants provided their written informed consent to participate in this study.

## Author contributions

MN: Data curation, Formal analysis, Writing – original draft, Conceptualization. EK: Conceptualization, Data curation, Writing – review & editing. AS: Funding acquisition, Supervision, Validation, Writing – review & editing. MB: Data curation, Formal analysis, Supervision, Validation, Visualization, Writing – original draft, Writing – review & editing.

## Footnotes

^1^ https://adni.loni.usc.edu/

^3^ https://www.mrtrix.org/

^4^ https://github.com/ANTsX/ANTs

^5^ https://dipy.org/
