# Supplementary material for: Exploring white matter microstructural alterations in mild cognitive impairment: a multimodal diffusion MRI investigation utilizing diffusion kurtosis and free-water imaging
Source: Front Neurosci. 2024 Aug 7;18:1440653. doi: 10.3389/fnins.2024.1440653 (PMC11335656; doi:10.3389/fnins.2024.1440653)
Supplement: Supplementary file 1 [file Table_1.DOCX]

Supplementary Material

**Sup. Table 1**

|  | ***f*-index** | | |
| --- | --- | --- | --- |
|  | **CN < MCI** | | |
|  | **t-stats** | | **effect-size** |
| **JHU Atlas** | **Vol (%)** | **<t>** | **g**max |
| Anterior thalamic radiation L | 9.02 | 2.202 | 0.827 |
| Anterior thalamic radiation R | 19.04 | 2.146 | 0.810 |
| Cortical spinal tract L | 6.25 | 2.002 | 0.631 |
| Cortical spinal tract R | 17.03 | 1.990 | 0.662 |
| Cingulum cingulate gyrus L | 12.71 | 2.018 | 0.762 |
| Cingulum cingulate gyrus R | 7.43 | 2.208 | 0.680 |
| Cingulum hippocampus L | 2.19 * | 1.766 | 0.524 |
| Cingulum hippocampus R | 21.62 | 2.563 | 0.798 |
| Forceps major | 21.15 | 2.266 | 0.735 |
| Forceps minor | 21.69 | 2.403 | 0.877 |
| Inferior fronto-occipital fasciculus L | 9.29 | 2.109 | 0.761 |
| Inferior fronto-occipital fasciculus R | 23.84 | 2.220 | 0.858 |
| Inferior longitudinal fasciculus L | 5.06 | 2.006 | 0.845 |
| Inferior longitudinal fasciculus R | 28.33 | 2.305 | 0.795 |
| Superior longitudinal fasciculus L | 9.00 | 2.097 | 0.905 |
| Superior longitudinal fasciculus R | 7.93 | 2.013 | 0.674 |
| Uncinate fasciculus L | 9.77 | 2.146 | 0.793 |
| Uncinate fasciculus R | 33.49 | 2.353 | 0.861 |
| Superior longitudinal fasciculus temporal L | 11.96 | 1.948 | 0.785 |
| Superior longitudinal fasciculus temporal R | 9.51 | 1.926 | 0.674 |
|  | **t-stats** | | **effect-size** |
| **ICBM81 Atlas** | **Vol (%)** | **<t>** | **g**max |
| Middle cerebellar peduncle | 10.31 * | 1.991 | 0.605 |
| Pontine crossing tract | 16.53 * | 1.993 | 0.559 |
| Genu of corpus callosum | 43.78 | 2.378 | 0.825 |
| Body of corpus callosum | 23.37 * | 2.066 | 0.519 |
| Splenium of corpus callosum | 46.75 | 2.394 | 0.735 |
| Corticospinal tract R | 10.21 * | 1.995 | 0.511 |
| Corticospinal tract L | 9.42 * | 2.018 | 0.498 |
| Medial lemniscus R | 17.39 * | 1.868 | 0.513 |
| Medial lemniscus L | 21.03 * | 2.056 | 0.441 |
| Inferior cerebellar peduncle R | 7.54 * | 1.790 | 0.428 |
| Inferior cerebellar peduncle L | 13.53 * | 2.044 | 0.467 |
| Superior cerebellar peduncle R | 25.00 | 2.465 | 0.646 |
| Superior cerebellar peduncle L | 25.00 * | 2.346 | 0.593 |
| Cerebral peduncle R | 56.23 | 2.042 | 0.662 |
| Cerebral peduncle L | 9.66 * | 1.904 | 0.578 |
| Anterior limb of internal capsule R | 17.94 | 2.224 | 0.629 |
| Posterior limb of internal capsule R | 13.91 * | 1.921 | 0.550 |
| Posterior limb of internal capsule L | 5.86 * | 1.828 | 0.525 |
| Retrolenticular part of internal capsule R | 5.61 * | 1.826 | 0.411 |
| Retrolenticular part of internal capsule L | 11.22 * | 2.028 | 0.538 |
| Anterior corona radiata R | 18.32 | 2.332 | 0.858 |
| Anterior corona radiata L | 4.82 | 2.096 | 0.672 |
| Superior corona radiata R | 16.99 * | 1.945 | 0.565 |
| Superior corona radiata L | 12.64 * | 1.977 | 0.577 |
| Posterior corona radiata R | 8.83 * | 1.975 | 0.412 |
| Posterior corona radiata L | 2.45 * | 1.775 | 0.383 |
| Posterior thalamic radiation R | 24.85 | 2.204 | 0.622 |
| Posterior thalamic radiation L | 10.23 * | 2.002 | 0.588 |
| Sagittal stratum R | 40.31 | 2.383 | 0.757 |
| External capsule R | 14.49 * | 2.082 | 0.585 |
| External capsule L | 9.00 * | 2.137 | 0.546 |
| Cingulum (cingulate gyrus) R | 8.63 * | 2.226 | 0.552 |
| Cingulum (cingulate gyrus) L | 18.36 | 1.979 | 0.762 |
| Cingulum (hippocampus) R | 43.93 | 2.634 | 0.798 |
| Cingulum (hippocampus) L | 0.78 * | 1.632 | 0.302 |
| Fornix (cres) / Stria terminalis R | 24.38 * | 2.248 | 0.524 |
| Fornix (cres) / Stria terminalis L | 8.44 * | 1.809 | 0.509 |
| Superior longitudinal fasciculus R | 7.07 * | 1.900 | 0.519 |
| Superior longitudinal fasciculus L | 17.11 | 1.915 | 0.690 |
| Uncinate fasciculus R | 74.21 | 2.388 | 0.614 |
| Uncinate fasciculus L | 9.31 * | 2.054 | 0.527 |
| Tapetum R | 13.09 * | 2.339 | 0.544 |
| Tapetum L | 5.83 * | 2.398 | 0.434 |

Complete results for the *f*-index (See Figure 1 panel (a)).

* medium and/or low effect-size (|g| < 0.61).

Vol (%): Percentage of the cluster's volume within the respective white matter area, <t>: Mean t value within the cluster; *g*_max_: Maximum *g* value within the cluster.

**Sup. Table 2**

|  | **fw-FA** | | | | | |
| --- | --- | --- | --- | --- | --- | --- |
|  | **CN < MCI** | | | **CN > MCI** | | |
|  | **t-stats** | | **effect-size** | **t-stats** | | **effect-size** |
| **JHU Atlas** | **Vol (%)** | **<t>** | **g_max_** | **Vol (%)** | **<t>** | **g_max_** |
| Anterior thalamic radiation L | - | - | - | 4.52 | -2.347 | -0.637 |
| Anterior thalamic radiation R | - | - | - | 2.31 | -2.587 | -0.690 |
| Cortical spinal tract L | - | - | - | 0.86 * | -2.283 | -0.500 |
| Cortical spinal tract R | - | - | - | 0.47 * | -2.208 | -0.365 |
| Cingulum cingulate gyrus L | 0.35 * | 2.292 | 0.569 | 1.07 * | -2.202 | -0.530 |
| Cingulum cingulate gyrus R | - | - | - | 0.34 * | -2.139 | -0.530 |
| Cingulum hippocampus L | - | - | - | 5.57 | -2.521 | -0.642 |
| Forceps major | 2.70 | 2.583 | 1.057 | 1.78 * | -2.277 | -0.523 |
| Forceps minor | - | - | - | 1.55 | -2.276 | -0.624 |
| Inferior fronto-occipital fasciculus L | 0.96 | 2.765 | 1.057 | 2.48 | -2.500 | -0.704 |
| Inferior fronto-occipital fasciculus R | 0.30 | 2.325 | 0.804 | 1.66 | -2.526 | -0.695 |
| Inferior longitudinal fasciculus L | 1.05 | 2.663 | 1.036 | 3.63 | -2.506 | -0.876 |
| Inferior longitudinal fasciculus R | - | - | - | 1.65 | -2.572 | -0.695 |
| Superior longitudinal fasciculus L | - | - | - | 3.06 | -2.445 | -0.778 |
| Uncinate fasciculus L | - | - | - | 0.75 | -2.404 | -0.632 |
| Uncinate fasciculus R | - | - | - | 1.20 * | -2.554 | -0.606 |
| Superior longitudinal fasciculus temporal L | - | - | - | 5.62 | -2.422 | -0.712 |
|  | **t-stats** | | **effect-size** | **t-stats** | | **effect-size** |
| **ICBM81 Atlas** | **Vol (%)** | **<t>** | **g_max_** | **Vol (%)** | **<t>** | **g_max_** |
| Middle cerebellar peduncle | 0.95 | 2.554 | 0.709 | - | - | - |
| Genu of corpus callosum | - | - | - | 2.70 * | -2.181 | -0.560 |
| Body of corpus callosum | 0.86 * | 2.219 | 0.569 | 1.76 * | -2.247 | -0.474 |
| Splenium of corpus callosum | - | - | - | 8.93 * | -2.167 | -0.523 |
| Inferior cerebellar peduncle R | 4.24 * | 2.642 | 0.456 | - | - | - |
| Superior cerebellar peduncle R | 0.40 * | 2.541 | 0.453 | - | - | - |
| Anterior limb of internal capsule L | - | - | - | 1.52 * | -2.564 | -0.557 |
| Retrolenticular part of internal capsule R | - | - | - | 0.28 * | -2.560 | -0.525 |
| Retrolenticular part of internal capsule L | - | - | - | 1.62 * | -2.460 | -0.596 |
| Anterior corona radiata R | - | - | - | 3.90 | -2.291 | -0.624 |
| Anterior corona radiata L | - | - | - | 1.90 * | -2.221 | -0.551 |
| Superior corona radiata R | - | - | - | 0.61 * | -2.231 | -0.299 |
| Superior corona radiata L | - | - | - | 4.17 * | -2.351 | -0.497 |
| Posterior corona radiata R | - | - | - | 5.12 * | -2.161 | -0.551 |
| Posterior corona radiata L | - | - | - | 0.35 * | -2.260 | -0.508 |
| Posterior thalamic radiation L | - | - | - | 6.06 | -2.599 | -0.623 |
| Sagittal stratum R | - | - | - | 10.82 | -2.590 | -0.695 |
| Sagittal stratum L | - | - | - | 3.23 * | -2.405 | -0.555 |
| Cingulum (cingulate gyrus) L | 0.98 * | 2.273 | 0.567 | - | - | - |
| Cingulum (hippocampus) L | - | - | - | 9.52 | -2.521 | -0.613 |
| Fornix (cres) / Stria terminalis R | - | - | - | 11.30 | -3.017 | -0.647 |
| Fornix (cres) / Stria terminalis L | - | - | - | 4.09 | -2.486 | -0.606 |
| Superior longitudinal fasciculus L | - | - | - | 5.22 | -2.372 | -0.778 |

Complete results for the fw-FA metric (See Figure 1 panel (b)).

* medium and/or low effect-size (|g| < 0.61).

Vol (%): Percentage of the cluster's volume within the respective white matter area, <t>: Mean t value within the cluster; *g*_max_: Maximum *g* value within the cluster.

**Sup. Table 3**

|  | **MKT** | | | | | |
| --- | --- | --- | --- | --- | --- | --- |
|  | **CN < MCI** | | | **CN > MCI** | | |
|  | **t-stats** | | **effect-size** | **t-stats** | | **effect-size** |
| **JHU Atlas** | **Vol (%)** | **<t>** | **g_max_** | **Vol (%)** | **<t>** | **g_max_** |
| Anterior thalamic radiation L | - | - | - | 13.67 * | -2.243 | -0.549 |
| Anterior thalamic radiation R | - | - | - | 12.65 * | -2.267 | -0.467 |
| Cortical spinal tract L | - | - | - | 17.97 * | -2.157 | -0.479 |
| Cortical spinal tract R | 0.24 | 2.552 | 0.697 | 20.13 * | -2.622 | -0.516 |
| Cingulum cingulate gyrus L | - | - | - | 10.34 * | -2.185 | -0.553 |
| Cingulum cingulate gyrus R | - | - | - | 6.41 * | -2.051 | -0.454 |
| Forceps major | 0.97 | 2.623 | 0.640 | 3.52 * | -2.032 | -0.529 |
| Forceps minor | 0.22 | 2.663 | 0.739 | 4.95* | -2.068 | -0.521 |
| Inferior fronto-occipital fasciculus L | 0.38 | 2.588 | 0.620 | 16.43 | -2.220 | -0.708 |
| Inferior fronto-occipital fasciculus R | - | - | - | 17.36 * | -2.135 | -0.537 |
| Inferior longitudinal fasciculus L | 0.58 | 2.652 | 0.849 | 14.73 | -2.347 | -0.755 |
| Inferior longitudinal fasciculus R | - | - | - | 14.36 * | -2.122 | -0.537 |
| Superior longitudinal fasciculus L | - | - | - | 18.91 | -2.539 | -0.747 |
| Superior longitudinal fasciculus R | - | - | - | 20.89 | -2.542 | -0.732 |
| Uncinate fasciculus L | - | - | - | 10.20 * | -2.100 | -0.484 |
| Uncinate fasciculus R | 0.28 | 2.587 | 0.660 | 8.41 * | -2.204 | -0.425 |
| Superior longitudinal fasciculus temporal L | - | - | - | 31.72 | -2.556 | -0.747 |
| Superior longitudinal fasciculus temporal R | - | - | - | 35.23 | -2.574 | -0.732 |
|  | **t-stats** | | **effect-size** | **t-stats** | | **effect-size** |
| **ICBM81 Atlas** | **Vol (%)** | **<t>** | **g_max_** | **Vol (%)** | **<t>** | **g_max_** |
| Genu of corpus callosum | - | - | - | 2.18 * | -1.888 | -0.384 |
| Body of corpus callosum | - | - | - | 5.37 * | -2.083 | -0.360 |
| Splenium of corpus callosum | - | - | - | 16.08 | -2.435 | -0.661 |
| Anterior limb of internal capsule R | - | - | - | 8.57 * | -2.532 | -0.467 |
| Anterior limb of internal capsule L | - | - | - | 15.71 * | -2.287 | -0.458 |
| Posterior limb of internal capsule R | 0.77 | 2.492 | 0.649 | 1.86 * | -1.841 | -0.327 |
| Posterior limb of internal capsule L | - | - | - | 1.01 * | -1.856 | -0.409 |
| Retrolenticular part of internal capsule R | - | - | - | 24.53 * | -1.997 | -0.423 |
| Retrolenticular part of internal capsule L | - | - | - | 10.61 * | -1.948 | -0.479 |
| Anterior corona radiata R | - | - | - | 43.01 * | -2.109 | -0.517 |
| Anterior corona radiata L | - | - | - | 37.56 * | -2.109 | -0.463 |
| Superior corona radiata R | - | - | - | 65.49 * | -2.427 | -0.497 |
| Superior corona radiata L | - | - | - | 57.38 * | -2.213 | -0.567 |
| Posterior corona radiata R | - | - | - | 56.63 * | -2.691 | -0.568 |
| Posterior corona radiata L | - | - | - | 43.86 * | -2.232 | -0.499 |
| Posterior thalamic radiation R | - | - | - | 47.43 * | -2.103 | -0.489 |
| Posterior thalamic radiation L | - | - | - | 28.23 | -2.189 | -0.697 |
| Sagittal stratum R | - | - | - | 31.64 * | -2.191 | -0.537 |
| Sagittal stratum L | - | - | - | 18.15 * | -2.219 | -0.579 |
| External capsule R | - | - | - | 5.08 * | -2.011 | -0.413 |
| External capsule L | - | - | - | 0.77 * | -2.175 | -0.349 |
| Cingulum (cingulate gyrus) R | - | - | - | 3.03 * | -1.970 | -0.375 |
| Cingulum (cingulate gyrus) L | - | - | - | 7.78 * | -2.075 | -0.520 |
| Fornix (cres) / Stria terminalis R | - | - | - | 3.83 * | -1.820 | -0.288 |
| Superior longitudinal fasciculus R | - | - | - | 56.86 | -2.564 | -0.705 |
| Superior longitudinal fasciculus L | - | - | - | 52.94 | -2.570 | -0.637 |
| Superior fronto-occipital fasciculus R | - | - | - | 72.19 | -2.654 | -0.665 |
| Superior fronto-occipital fasciculus L | - | - | - | 74.36 | -2.880 | -0.649 |
| Tapetum R | - | - | - | 11.24 | -2.150 | -0.422 |

Complete results for the MKT (See Figure 2 panel (a)).

* medium and/or low effect-size (|g| < 0.61).

Vol (%): Percentage of the cluster's volume within the respective white matter area, <t>: Mean t value within the cluster; *g*_max_: Maximum *g* value within the cluster.

**Sup. Table 4**

|  | **KFA** | | |
| --- | --- | --- | --- |
|  | **CN > MCI** | | |
|  | **t-stats** | | **effect-size** |
| **JHU Atlas** | **Vol (%)** | **<t>** | **g_max_** |
| Anterior thalamic radiation L | 11.08 | -2.069 | -0.717 |
| Anterior thalamic radiation R | 14.61 | -2.318 | -0.934 |
| Cortical spinal tract L | 3.15 | -2.169 | -0.626 |
| Cortical spinal tract R | 7.69 | -2.290 | -0.630 |
| Cingulum cingulate gyrus L | 6.67 | -2.137 | -0.958 |
| Cingulum cingulate gyrus R | 2.25 | -2.152 | -0.649 |
| Cingulum hippocampus R | 9.48 | -2.824 | -0.845 |
| Forceps major | 6.69 | -2.461 | -0.690 |
| Forceps minor | 17.93 | -2.411 | -0.958 |
| Inferior fronto-occipital fasciculus L | 10.43 | -2.123 | -0.788 |
| Inferior fronto-occipital fasciculus R | 14.03 | -2.312 | -0.934 |
| Inferior longitudinal fasciculus L | 2.09 | -2.315 | -0.685 |
| Inferior longitudinal fasciculus R | 12.78 | -2.311 | -0.748 |
| Superior longitudinal fasciculus L | 3.41 | -2.271 | -0.685 |
| Superior longitudinal fasciculus R | 2.46 | -2.269 | -0.614 |
| Uncinate fasciculus L | 17.83 | -2.125 | -0.788 |
| Uncinate fasciculus R | 30.53 | -2.425 | -0.903 |
| Superior longitudinal fasciculus temporal L | 5.75 | -2.250 | -0.685 |
| Superior longitudinal fasciculus temporal R | 4.72 | -2.343 | -0.614 |
|  | **t-stats** | | **effect-size** |
| **ICBM81 Atlas** | **Vol (%)** | **<t>** | **g_max_** |
| Middle cerebellar peduncle | 0.30 * | -2.506 | -0.541 |
| Pontine crossing tract | 23.37 * | -2.234 | -0.532 |
| Genu of corpus callosum | 33.34 | -2.342 | -0.898 |
| Body of corpus callosum | 1.65 * | -1.987 | -0.432 |
| Splenium of corpus callosum | 21.39 | -2.441 | -0.709 |
| Corticospinal tract R | 4.55 * | -2.182 | -0.454 |
| Corticospinal tract L | 7.01 * | -2.060 | -0.509 |
| Medial lemniscus R | 30.14 | -2.341 | -0.664 |
| Medial lemniscus L | 29.47 * | -2.360 | -0.498 |
| Inferior cerebellar peduncle R | 0.62 * | -1.779 | -0.252 |
| Inferior cerebellar peduncle L | 0.41 * | -1.772 | -0.255 |
| Superior cerebellar peduncle R | 15.83 | -2.429 | -0.639 |
| Superior cerebellar peduncle L | 5.34 * | -2.058 | -0.392 |
| Cerebral peduncle R | 20.06 | -2.352 | -0.665 |
| Cerebral peduncle L | 5.62 | -2.007 | -0.628 |
| Anterior limb of internal capsule R | 13.96 | -2.438 | -0.647 |
| Anterior limb of internal capsule L | 15.37 * | -2.072 | -0.545 |
| Posterior limb of internal capsule R | 7.88 | -2.277 | -0.630 |
| Posterior limb of internal capsule L | 20.34 | -1.998 | -0.656 |
| Retrolenticular part of internal capsule L | 11.46 | -2.071 | -0.642 |
| Anterior corona radiata R | 22.69 | -2.607 | -0.934 |
| Anterior corona radiata L | 13.65 | -2.194 | -0.780 |
| Superior corona radiata R | 6.36 * | -2.314 | -0.358 |
| Superior corona radiata L | 0.27 * | -2.040 | -0.277 |
| Posterior corona radiata R | 5.04 * | -2.028 | -0.495 |
| Posterior thalamic radiation R | 4.25 * | -2.021 | -0.363 |
| Posterior thalamic radiation L | 3.32 | -2.322 | -0.627 |
| Sagittal stratum R | 25.27 | -2.399 | -0.729 |
| Sagittal stratum L | 0.76 * | -1.808 | -0.474 |
| External capsule R | 9.86 | -2.192 | -0.623 |
| External capsule L | 18.20 | -2.162 | -0.788 |
| Cingulum (cingulate gyrus) R | 5.42 * | -2.208 | -0.511 |
| Cingulum (cingulate gyrus) L | 5.49 * | -2.096 | -0.536 |
| Cingulum (hippocampus) R | 25.65 | -2.738 | -0.845 |
| Fornix (cres) / Stria terminalis R | 17.44 | -3.071 | -0.705 |
| Superior longitudinal fasciculus R | 2.45 * | -2.434 | -0.389 |
| Superior longitudinal fasciculus L | 10.86 * | -2.240 | -0.579 |
| Uncinate fasciculus R | 27.89 * | -2.278 | -0.576 |
| Uncinate fasciculus L | 2.13 * | -1.776 | -0.367 |

Complete results for the KFA (See Figure 2 panel (b)).

* medium and/or low effect-size (|g| < 0.61).

Vol (%): Percentage of the cluster's volume within the respective white matter area, <t>: Mean t value within the cluster; *g*_max_: Maximum *g* value within the cluster.

**Sup. Table 5**

|  | **MK** | | | | | |
| --- | --- | --- | --- | --- | --- | --- |
|  | **CN < MCI** | | | **CN > MCI** | | |
|  | **t-stats** | | **effect-size** | **t-stats** | | **effect-size** |
| **JHU Atlas** | **Vol (%)** | **<t>** | **g_max_** | **Vol (%)** | **<t>** | **g_max_** |
| Anterior thalamic radiation L | - | - | - | 14.33 * | -2.231 | -0.588 |
| Anterior thalamic radiation R | 1.38 | 2.508 | 0.708 | 13.00 * | -2.232 | -0.501 |
| Cortical spinal tract L | - | - | - | 20.04 | -2.306 | -0.612 |
| Cortical spinal tract R | 0.54 | 2.588 | 0.774 | 20.24 * | -2.673 | -0.537 |
| Cingulum cingulate gyrus L | - | - | - | 11.70 * | -2.193 | -0.580 |
| Cingulum cingulate gyrus R | - | - | - | 7.11 * | -2.143 | -0.436 |
| Forceps major | 0.90 | 2.660 | 0.641 | 4.28 | -1.949 | -0.617 |
| Forceps minor | 0.73 | 2.710 | 0.784 | 5.10 * | -2.022 | -0.523 |
| Inferior fronto-occipital fasciculus L | 0.48 | 2.668 | 0.634 | 20.67 | -2.224 | -0.698 |
| Inferior fronto-occipital fasciculus R | - | - | - | 19.25 | -2.203 | -0.640 |
| Inferior longitudinal fasciculus L | 0.79 | 2.639 | 0.827 | 20.19 | -2.347 | -0.737 |
| Inferior longitudinal fasciculus R | - | - | - | 14.80 * | -2.202 | -0.585 |
| Superior longitudinal fasciculus L | - | - | - | 20.04 | -2.509 | -0.737 |
| Superior longitudinal fasciculus R | - | - | - | 20.68 | -2.523 | -0.746 |
| Uncinate fasciculus L | - | - | - | 9.54 * | -2.030 | -0.467 |
| Uncinate fasciculus R | 0.44 | 2.724 | 0.708 | 7.80 * | -2.154 | -0.400 |
| Superior longitudinal fasciculus temporal L | - | - | - | 33.98 | -2.533 | -0.737 |
| Superior longitudinal fasciculus temporal R | - | - | - | 35.90 | -2.581 | -0.746 |
|  | **t-stats** | | **effect-size** | **t-stats** | | **effect-size** |
| **ICBM81 Atlas** | **Vol (%)** | **<t>** | **g_max_** | **Vol (%)** | **<t>** | **g_max_** |
| Genu of corpus callosum | 1.80 * | 2.622 | 0.564 | 3.76 * | -1.918 | -0.375 |
| Body of corpus callosum | - | - | - | 10.20 * | -2.173 | -0.481 |
| Splenium of corpus callosum | - | - | - | 19.49 | -2.594 | -0.665 |
| Anterior limb of internal capsule R | 4.43 * | 2.376 | 0.578 | 8.32 * | -2.441 | -0.484 |
| Anterior limb of internal capsule L | - | - | - | 19.42 * | -2.354 | -0.468 |
| Posterior limb of internal capsule R | 3.17 | 2.429 | 0.753 | 2.45 * | -1.876 | -0.377 |
| Posterior limb of internal capsule L | - | - | - | 2.40 * | -1.931 | -0.524 |
| Retrolenticular part of internal capsule R | - | - | - | 57.97 | -2.293 | -0.640 |
| Retrolenticular part of internal capsule L | - | - | - | 18.64 * | -2.086 | -0.549 |
| Anterior corona radiata R | - | - | - | 43.71 * | -2.088 | -0.523 |
| Anterior corona radiata L | - | - | - | 36.03 * | -2.031 | -0.441 |
| Superior corona radiata R | - | - | - | 64.39 * | -2.445 | -0.552 |
| Superior corona radiata L | - | - | - | 62.85 | -2.257 | -0.632 |
| Posterior corona radiata R | - | - | - | 57.89 * | -2.890 | -0.587 |
| Posterior corona radiata L | - | - | - | 43.81 * | -2.295 | -0.542 |
| Posterior thalamic radiation R | - | - | - | 50.28 | -2.226 | -0.683 |
| Posterior thalamic radiation L | - | - | - | 45.45 | -2.135 | -0.698 |
| Sagittal stratum R | - | - | - | 36.85 | -2.146 | -0.653 |
| Sagittal stratum L | - | - | - | 32.99 | -2.390 | -0.623 |
| External capsule R | - | - | - | 4.24 * | -1.871 | -0.505 |
| Cingulum (cingulate gyrus) R | - | - | - | 3.59 * | -2.036 | -0.424 |
| Cingulum (cingulate gyrus) L | - | - | - | 8.03 * | -2.122 | -0.550 |
| Fornix (cres) / Stria terminalis L | - | - | - | 3.29 * | -1.861 | -0.415 |
| Superior longitudinal fasciculus R | - | - | - | 57.21 | -2.569 | -0.724 |
| Superior longitudinal fasciculus L | - | - | - | 53.93 | -2.558 | -0.618 |
| Superior fronto-occipital fasciculus R | - | - | - | 73.18 * | -2.549 | -0.501 |
| Superior fronto-occipital fasciculus L | - | - | - | 78.11 * | -3.005 | -0.568 |
| Tapetum R | - | - | - | 17.95 * | -2.406 | -0.524 |
| Tapetum L | - | - | - | 5.00 * | -1.931 | -0.336 |

Complete results for the MK (See Figure 2 panel (c)).

* medium and/or low effect-size (|g| < 0.61).

Vol (%): Percentage of the cluster's volume within the respective white matter area, <t>: Mean t value within the cluster; *g*_max_: Maximum *g* value within the cluster.

**Sup. Table 6**

|  | **AK** | | | | | |
| --- | --- | --- | --- | --- | --- | --- |
|  | **CN < MCI** | | | **CN > MCI** | | |
|  | **t-stats** | | **effect-size** | **t-stats** | | **effect-size** |
| **JHU Atlas** | **Vol (%)** | **<t>** | **g_max_** | **Vol (%)** | **<t>** | **g_max_** |
| Anterior thalamic radiation L | 0.38 * | 2.567 | 0.572 | 5.05 * | -2.259 | -0.498 |
| Anterior thalamic radiation R | - | - | - | 3.32 * | -2.128 | -0.474 |
| Cortical spinal tract L | - | - | - | 2.54 * | -2.114 | -0.540 |
| Cortical spinal tract R | - | - | - | 7.60 | -2.270 | -0.681 |
| Cingulum cingulate gyrus L | - | - | - | 3.23 * | -2.016 | -0.499 |
| Cingulum cingulate gyrus R | - | - | - | 3.26 * | -2.088 | -0.517 |
| Forceps major | - | - | - | 1.66 * | -2.217 | -0.450 |
| Forceps minor | - | - | - | 2.13 * | -2.039 | -0.510 |
| Inferior fronto-occipital fasciculus L | - | - | - | 5.80 | -2.253 | -0.675 |
| Inferior fronto-occipital fasciculus R | - | - | - | 5.33 * | -2.217 | -0.520 |
| Inferior longitudinal fasciculus L | - | - | - | 5.66 * | -2.364 | -0.691 |
| Inferior longitudinal fasciculus R | 0.29 | 2.637 | 0.736 | 2.32 * | -2.347 | -0.511 |
| Superior longitudinal fasciculus L | - | - | - | 10.56 | -2.463 | -0.846 |
| Superior longitudinal fasciculus R | - | - | - | 14.53 | -2.521 | -0.710 |
| Uncinate fasciculus L | - | - | - | 3.67 * | -2.231 | -0.478 |
| Uncinate fasciculus R | - | - | - | 3.35 * | -2.148 | -0.466 |
| Superior longitudinal fasciculus temporal L | - | - | - | 16.55 | -2.475 | -0.846 |
| Superior longitudinal fasciculus temporal R | - | - | - | 23.48 | -2.680 | -0.710 |
|  | **t-stats** | | **effect-size** | **t-stats** | | **effect-size** |
| **ICBM81 Atlas** | **Vol (%)** | **<t>** | **g_max_** | **Vol (%)** | **<t>** | **g_max_** |
| Genu of corpus callosum | - | - | - | 0.53 * | -1.946 | -0.373 |
| Body of corpus callosum | - | - | - | 0.72 * | -2.048 | -0.320 |
| Splenium of corpus callosum | - | - | - | 3.82 * | -2.173 | -0.562 |
| Cerebral peduncle L | 1.40 * | 2.549 | 0.572 | - | - | - |
| Anterior limb of internal capsule R | - | - | - | 1.05 * | -1.822 | -0.235 |
| Anterior limb of internal capsule L | - | - | - | 1.72 * | -2.166 | -0.407 |
| Retrolenticular part of internal capsule R | - | - | - | - | - | - |
| Anterior corona radiata R | - | - | - | 10.28 * | -2.127 | -0.474 |
| Anterior corona radiata L | - | - | - | 20.29 * | -2.310 | -0.553 |
| Superior corona radiata R | - | - | - | 25.96 * | -2.215 | -0.565 |
| Superior corona radiata L | - | - | - | 19.69 | -2.277 | -0.620 |
| Posterior corona radiata R | - | - | - | 32.00 * | -2.333 | -0.489 |
| Posterior corona radiata L | - | - | - | 19.01 * | -2.258 | -0.516 |
| Posterior thalamic radiation R | - | - | - | 17.40 * | -2.215 | -0.520 |
| Posterior thalamic radiation L | - | - | - | 11.64 * | -2.185 | -0.544 |
| Sagittal stratum R | - | - | - | 4.49 * | -2.425 | -0.282 |
| External capsule R | - | - | - | 2.37 * | -2.001 | -0.452 |
| Cingulum (cingulate gyrus) R | - | - | - | 1.58 * | -1.924 | -0.401 |
| Cingulum (cingulate gyrus) L | - | - | - | 3.64 * | -2.024 | -0.382 |
| Superior longitudinal fasciculus R | - | - | - | 37.67 | -2.590 | -0.686 |
| Superior longitudinal fasciculus L | - | - | - | 29.48 | -2.425 | -0.741 |
| Superior fronto-occipital fasciculus R | - | - | - | 1.38 * | -1.761 | -0.233 |
| Superior fronto-occipital fasciculus L | - | - | - | 27.42 * | -2.255 | -0.420 |

Complete results for the AK (See Figure 2 panel (d)).

* medium and/or low effect-size (|g| < 0.61).

Vol (%): Percentage of the cluster's volume within the respective white matter area, <t>: Mean t value within the cluster; *g*_max_: Maximum *g* value within the cluster.

**Sup. Table 7**

|  | **RK** | | | | | |
| --- | --- | --- | --- | --- | --- | --- |
|  | **CN < MCI** | | | **CN > MCI** | | |
|  | **t-stats** | | **effect-size** | **t-stats** | | **effect-size** |
| **JHU Atlas** | **Vol (%)** | **<t>** | **g_max_** | **Vol (%)** | **<t>** | **g_max_** |
| Anterior thalamic radiation L | 0.37 | 2.413 | 0.671 | 8.00 * | -2.108 | -0.573 |
| Anterior thalamic radiation R | 0.93 | 2.753 | 0.934 | 5.59 * | -2.052 | -0.454 |
| Cortical spinal tract L | - | - | - | 7.57 * | -2.056 | -0.576 |
| Cortical spinal tract R | 0.51 | 2.676 | 0.787 | 9.89 * | -2.232 | -0.559 |
| Cingulum cingulate gyrus L | - | - | - | 11.65 * | -2.074 | -0.571 |
| Cingulum cingulate gyrus R | - | - | - | 6.27 * | -2.122 | -0.547 |
| Cingulum hippocampus R | - | - | - | 6.45 * | -2.004 | -0.462 |
| Forceps major | 1.90 | 2.607 | 0.831 | 3.32 * | -1.989 | -0.595 |
| Forceps minor | 0.57 | 2.864 | 0.827 | 2.45 * | -1.971 | -0.581 |
| Inferior fronto-occipital fasciculus L | 0.83 | 2.751 | 0.839 | 13.70 | -2.232 | -0.705 |
| Inferior fronto-occipital fasciculus R | - | - | - | 13.19 | -2.183 | -0.809 |
| Inferior longitudinal fasciculus L | 1.30 | 2.657 | 0.828 | 17.14 | -2.345 | -0.783 |
| Inferior longitudinal fasciculus R | - | - | - | 11.31 | -2.093 | -0.606 |
| Superior longitudinal fasciculus L | - | - | - | 15.26 | -2.266 | -0.723 |
| Superior longitudinal fasciculus R | - | - | - | 10.01 | -2.112 | -0.641 |
| Uncinate fasciculus L | - | - | - | 2.60 * | -1.891 | -0.536 |
| Uncinate fasciculus R | 0.74 | 2.719 | 0.730 | 3.73 * | -2.087 | -0.416 |
| Superior longitudinal fasciculus temporal L | - | - | - | 26.37 | -2.307 | -0.723 |
| Superior longitudinal fasciculus temporal R | - | - | - | 19.73 | -2.089 | -0.622 |
|  | **t-stats** | | **effect-size** | **t-stats** | | **effect-size** |
| **ICBM81 Atlas** | **Vol (%)** | **<t>** | **g_max_** | **Vol (%)** | **<t>** | **g_max_** |
| Middle cerebellar peduncle | - | - | - | 1.25 * | -2.543 | -0.496 |
| Genu of corpus callosum | - | - | - | 4.75 * | -2.058 | -0.381 |
| Body of corpus callosum | - | - | - | 10.15 * | -2.114 | -0.547 |
| Splenium of corpus callosum | - | - | - | 23.97 | -2.345 | -0.627 |
| Anterior limb of internal capsule R | 1.21 | 2.471 | 0.651 | 1.85 * | -2.099 | -0.377 |
| Anterior limb of internal capsule L | 1.19 | 2.481 | 0.665 | 17.63 * | -2.094 | -0.430 |
| Posterior limb of internal capsule R | 6.79 | 2.395 | 0.734 | 1.04 * | -1.888 | -0.450 |
| Posterior limb of internal capsule L | 3.49 | 2.291 | 0.637 | - | - | - |
| Retrolenticular part of internal capsule R | - | - | - | 59.13 | -2.363 | -0.809 |
| Retrolenticular part of internal capsule L | - | - | - | 16.20 | -1.985 | -0.642 |
| Anterior corona radiata R | - | - | - | 14.49 * | -2.044 | -0.581 |
| Anterior corona radiata L | - | - | - | 10.48 * | -1.892 | -0.417 |
| Superior corona radiata R | - | - | - | 20.03 * | -2.099 | -0.521 |
| Superior corona radiata L | - | - | - | 21.26 * | -1.991 | -0.534 |
| Posterior corona radiata R | - | - | - | 44.50 | -2.401 | -0.643 |
| Posterior corona radiata L | - | - | - | 13.87 * | -2.068 | -0.539 |
| Posterior thalamic radiation R | - | - | - | 32.80 | -2.173 | -0.661 |
| Posterior thalamic radiation L | - | - | - | 31.25 | -2.146 | -0.704 |
| Sagittal stratum R | - | - | - | 35.77 | -1.999 | -0.636 |
| Sagittal stratum L | - | - | - | 26.94 | -2.348 | -0.705 |
| External capsule R | - | - | - | 2.98 * | -1.829 | -0.592 |
| Cingulum (cingulate gyrus) R | - | - | - | 5.04 * | -1.995 | -0.504 |
| Cingulum (cingulate gyrus) L | - | - | - | 7.56 * | -1.998 | -0.492 |
| Cingulum (hippocampus) R | - | - | - | 8.82 * | -2.032 | -0.431 |
| Fornix (cres) / Stria terminalis R | - | - | - | 1.51 * | -1.738 | -0.334 |
| Fornix (cres) / Stria terminalis L | - | - | - | 8.18 * | -1.918 | -0.541 |
| Superior longitudinal fasciculus R | - | - | - | 30.65 * | -2.046 | -0.552 |
| Superior longitudinal fasciculus L | - | - | - | 37.21 | -2.144 | -0.649 |
| Superior fronto-occipital fasciculus R | - | - | - | 10.26 * | -2.098 | -0.412 |
| Superior fronto-occipital fasciculus L | - | - | - | 62.13 * | -2.394 | -0.503 |
| Tapetum R | - | - | - | 18.96 * | -2.518 | -0.579 |
| Tapetum L | - | - | - | 6.00 * | -2.195 | -0.430 |

Complete results for the RK (See Figure 2 panel (e)).

* medium and/or low effect-size (|g| < 0.61).

Vol (%): Percentage of the cluster's volume within the respective white matter area, <t>: Mean t value within the cluster; *g*_max_: Maximum *g* value within the cluster.

**Sup. Table 8**

|  | **MSD** | | |
| --- | --- | --- | --- |
|  | **CN < MCI** | | |
|  | **t-stats** | | **effect-size** |
| **JHU Atlas** | **Vol (%)** | **<t>** | **g_max_** |
| Anterior thalamic radiation L | 28.87 | 2.176 | 0.777 |
| Anterior thalamic radiation R | 31.07 | 2.219 | 0.802 |
| Cortical spinal tract L | 26.17 * | 2.040 | 0.539 |
| Cortical spinal tract R | 32.29 | 2.359 | 0.698 |
| Cingulum cingulate gyrus L | 30.45 | 2.251 | 0.736 |
| Cingulum cingulate gyrus R | 20.61 | 2.222 | 0.671 |
| Cingulum hippocampus L | 16.76 * | 1.936 | 0.433 |
| Cingulum hippocampus R | 24.34 | 2.794 | 0.814 |
| Forceps major | 28.48 | 2.389 | 0.701 |
| Forceps minor | 33.75 | 2.608 | 0.841 |
| Inferior fronto-occipital fasciculus L | 34.25 | 2.190 | 0.777 |
| Inferior fronto-occipital fasciculus R | 42.38 | 2.478 | 0.841 |
| Inferior longitudinal fasciculus L | 20.60 | 2.125 | 0.787 |
| Inferior longitudinal fasciculus R | 44.61 | 2.601 | 0.864 |
| Superior longitudinal fasciculus L | 27.09 | 2.321 | 0.820 |
| Superior longitudinal fasciculus R | 23.28 | 2.189 | 0.703 |
| Uncinate fasciculus L | 39.63 | 2.227 | 0.750 |
| Uncinate fasciculus R | 46.44 | 2.707 | 0.804 |
| Superior longitudinal fasciculus temporal L | 40.16 | 2.287 | 0.714 |
| Superior longitudinal fasciculus temporal R | 32.91 | 2.137 | 0.703 |
|  | **t-stats** | | **effect-size** |
| **ICBM81 Atlas** | **Vol (%)** | **<t>** | **g_max_** |
| Middle cerebellar peduncle | 17.63 * | 2.020 | 0.440 |
| Pontine crossing tract | 18.27 * | 1.976 | 0.341 |
| Genu of corpus callosum | 56.28 | 2.779 | 0.781 |
| Body of corpus callosum | 45.25 * | 2.333 | 0.509 |
| Splenium of corpus callosum | 70.59 | 2.640 | 0.620 |
| Corticospinal tract R | 19.02 * | 2.342 | 0.514 |
| Corticospinal tract L | 14.89 * | 2.145 | 0.498 |
| Medial lemniscus R | 20.43 * | 1.873 | 0.436 |
| Medial lemniscus L | 24.46 * | 2.110 | 0.396 |
| Inferior cerebellar peduncle R | 17.25 * | 1.837 | 0.324 |
| Inferior cerebellar peduncle L | 15.91 * | 2.128 | 0.382 |
| Superior cerebellar peduncle R | 25.81 | 2.330 | 0.671 |
| Superior cerebellar peduncle L | 26.81 | 2.325 | 0.668 |
| Cerebral peduncle R | 46.66 | 2.246 | 0.668 |
| Cerebral peduncle L | 23.84 * | 1.952 | 0.514 |
| Anterior limb of internal capsule R | 26.35 * | 2.122 | 0.553 |
| Anterior limb of internal capsule L | 26.81 * | 1.913 | 0.426 |
| Posterior limb of internal capsule R | 9.54 * | 1.993 | 0.405 |
| Posterior limb of internal capsule L | 16.60 * | 1.838 | 0.506 |
| Retrolenticular part of internal capsule R | 44.97 * | 2.268 | 0.457 |
| Retrolenticular part of internal capsule L | 49.17 * | 2.283 | 0.526 |
| Anterior corona radiata R | 51.86 | 2.412 | 0.841 |
| Anterior corona radiata L | 48.19 | 2.014 | 0.645 |
| Superior corona radiata R | 54.67 | 2.316 | 0.639 |
| Superior corona radiata L | 48.52 | 2.174 | 0.641 |
| Posterior corona radiata R | 52.76 * | 2.306 | 0.462 |
| Posterior corona radiata L | 35.14 * | 2.191 | 0.418 |
| Posterior thalamic radiation R | 64.17 * | 2.490 | 0.596 |
| Posterior thalamic radiation L | 41.50 * | 2.134 | 0.569 |
| Sagittal stratum R | 66.65 | 2.634 | 0.660 |
| Sagittal stratum L | 12.10 * | 1.911 | 0.426 |
| External capsule R | 26.31 | 2.329 | 0.622 |
| External capsule L | 34.67 | 2.201 | 0.624 |
| Cingulum (cingulate gyrus) R | 26.17 * | 2.147 | 0.558 |
| Cingulum (cingulate gyrus) L | 36.46 | 2.181 | 0.736 |
| Cingulum (hippocampus) R | 47.49 | 2.900 | 0.814 |
| Cingulum (hippocampus) L | 24.07 * | 1.838 | 0.386 |
| Fornix (cres) / Stria terminalis R | 36.96 * | 2.397 | 0.436 |
| Fornix (cres) / Stria terminalis L | 11.64 * | 1.805 | 0.414 |
| Superior longitudinal fasciculus R | 51.23 * | 2.132 | 0.529 |
| Superior longitudinal fasciculus L | 69.83 | 2.341 | 0.663 |
| Superior fronto-occipital fasciculus R | 45.96 * | 1.827 | 0.240 |
| Superior fronto-occipital fasciculus L | 60.55 * | 2.013 | 0.345 |
| Uncinate fasciculus R | 76.84 | 3.186 | 0.666 |
| Uncinate fasciculus L | 32.98 * | 2.343 | 0.504 |
| Tapetum R | 17.95 * | 2.718 | 0.499 |
| Tapetum L | 7.67 * | 2.694 | 0.421 |

Complete results for the MSD (See Figure 3 panel (a)).

* medium and/or low effect-size (|g| < 0.61).

Vol (%): Percentage of the cluster's volume within the respective white matter area, <t>: Mean t value within the cluster; *g*_max_: Maximum *g* value within the cluster.

**Sup. Table 9**

|  | **MSK** | | |
| --- | --- | --- | --- |
|  | **CN > MCI** | | |
|  | **t-stats** | | **effect-size** |
| **JHU Atlas** | **Vol (%)** | **<t>** | **g_max_** |
| Anterior thalamic radiation L | 14.75 * | -2.159 | -0.504 |
| Anterior thalamic radiation R | 12.74 * | -2.168 | -0.441 |
| Cortical spinal tract L | 19.03 * | -2.146 | -0.461 |
| Cortical spinal tract R | 19.24 * | -2.566 | -0.545 |
| Cingulum cingulate gyrus L | 12.89 * | -2.189 | -0.561 |
| Cingulum cingulate gyrus R | 9.31 * | -2.028 | -0.456 |
| Forceps major | 9.59 * | -1.397 | -0.570 |
| Forceps minor | 7.74 * | -1.986 | -0.549 |
| Inferior fronto-occipital fasciculus L | 20.61 | -2.040 | -0.709 |
| Inferior fronto-occipital fasciculus R | 22.05 | -2.099 | -0.680 |
| Inferior longitudinal fasciculus L | 17.70 | -2.173 | -0.753 |
| Inferior longitudinal fasciculus R | 19.62 | -2.095 | -0.630 |
| Superior longitudinal fasciculus L | 20.02 | -2.478 | -0.700 |
| Superior longitudinal fasciculus R | 20.13 | -2.396 | -0.729 |
| Uncinate fasciculus L | 11.65 * | -2.080 | -0.489 |
| Uncinate fasciculus R | 9.98 * | -2.172 | -0.436 |
| Superior longitudinal fasciculus temporal L | 33.22 | -2.498 | -0.700 |
| Superior longitudinal fasciculus temporal R | 34.23 | -2.415 | -0.729 |
|  | **t-stats** | | **effect-size** |
| **ICBM81 Atlas** | **Vol (%)** | **<t>** | **g_max_** |
| Genu of corpus callosum | 10.99 * | -1.842 | -0.428 |
| Body of corpus callosum | 8.78 * | -2.046 | -0.371 |
| Splenium of corpus callosum | 32.43 * | -2.270 | -0.529 |
| Anterior limb of internal capsule R | 7.39 * | -2.331 | -0.424 |
| Anterior limb of internal capsule L | 20.87 * | -2.181 | -0.438 |
| Posterior limb of internal capsule L | 1.12 * | -1.825 | -0.385 |
| Retrolenticular part of internal capsule R | 39.84 * | -2.042 | -0.449 |
| Retrolenticular part of internal capsule L | 26.29 * | -1.972 | -0.528 |
| Anterior corona radiata R | 42.90 * | -2.057 | -0.549 |
| Anterior corona radiata L | 35.97 * | -2.011 | -0.489 |
| Superior corona radiata R | 57.21 * | -2.342 | -0.474 |
| Superior corona radiata L | 59.34 * | -2.136 | -0.528 |
| Posterior corona radiata R | 57.64 * | -2.594 | -0.530 |
| Posterior corona radiata L | 40.58 * | -2.081 | -0.466 |
| Posterior thalamic radiation R | 55.54 * | -2.055 | -0.556 |
| Posterior thalamic radiation L | 37.83 | -2.119 | -0.709 |
| Sagittal stratum R | 56.55 * | -2.126 | -0.576 |
| Sagittal stratum L | 20.93 * | -2.254 | -0.577 |
| External capsule L | 1.25 * | -2.186 | -0.434 |
| Cingulum (cingulate gyrus) R | 7.30 * | -1.953 | -0.431 |
| Cingulum (cingulate gyrus) L | 7.60 * | -2.138 | -0.528 |
| Fornix (cres) / Stria terminalis R | 19.31 * | -2.173 | -0.399 |
| Fornix (cres) / Stria terminalis L | 4.53 * | -1.749 | -0.359 |
| Superior longitudinal fasciculus R | 56.39 | -2.408 | -0.706 |
| Superior longitudinal fasciculus L | 54.88 | -2.519 | -0.651 |
| Superior fronto-occipital fasciculus R | 64.50 * | -2.388 | -0.441 |
| Superior fronto-occipital fasciculus L | 78.70 * | -2.668 | -0.504 |
| Tapetum R | 16.11 * | -2.252 | -0.459 |
| Tapetum L | 4.50 * | -2.044 | -0.358 |

Complete results for the MSK (See Figure 3 panel (b)).

* medium and/or low effect-size (|g| < 0.61).

Vol (%): Percentage of the cluster's volume within the respective white matter area, <t>: Mean t value within the cluster; *g*_max_: Maximum *g* value within the cluster.
